# Multifunctional Core–Shell Cobalt Oxide @ Carbon Nanodot Hybrid Conjugates for Imaging and Targeting A549 Cells

**DOI:** 10.1021/acsabm.5c00343

**Published:** 2025-06-05

**Authors:** Anitha Jayapalan, Frank Tukur, Mahsa Azami, Mengxin Liu, Jianjun Wei

**Affiliations:** The Department of Nanoscience, Joint School of Nanoscience and Nanoengineering, 14616 The University of North Carolina at Greensboro, 2907 E. Gate City Blvd, Greensboro, North Carolina 27401, United States

**Keywords:** active targeting, anticancer, receptor–ligand complex, A549 lung cancer cells, bioimaging

## Abstract

The advent of research using drug-delivery vehicles with nanoparticles (NPs) in treating and diagnosing lung cancer has created a potential development in cancer therapeutics. Using certain NP–based compositions, specifically hybrid NPs, the cancer cells could be detected with enhanced fluorescence ability and treated using targeted drug release while minimizing adverse effects. A modified microwave-based synthesis approach was used in this study to synthesize spherical core–shell hybrid cobalt oxide carbon nanodot (Co_3_O_4_@CND) NPs of a smaller size of around 20 nm. Four different targeting ligands—folic acid, heparin, PEGylated silica (SiO_2_), and transferrin—and the anticancer drug doxorubicin (DOX) were conjugated to the hybrid NPs, and their physicochemical characterizations were evaluated for their applications. The bioimaging, antioxidant, biocompatibility, cancer-targeting ability, and anticancerous specificity effect of the hybrid NPs were examined using A549 (lung cancer) cells and compared with CNDs, Co_3_O_4_ NPs, and the ligand-conjugated NPs. The Co_3_O_4_@CND NPs demonstrated high fluorescence from their synergistic properties, leading to a better bioimaging ability in human cells. The Co_3_O_4_@CND hybrid NP–transferrin–DOX composite targeted 50% of A549 cells with a much less adverse effect on EAhy926 (endothelial) cells at the same concentrations. Increased anticancer activity of the Co_3_O_4_@CNDs and improved biocompatibility were achieved via a receptor-mediated active targeting approach using specific ligands, proving the potential multifunctional applications such as bioimaging, antioxidant, and anticancer activity. After transferrin conjugation, the NP composite is more anticancerous to A549 and shows decreased toxicity to EAhy926 cells. The outcomes, while in the early stage, suggest that the Co_3_O_4_@CND hybrid NPs with ligand conjugation are a potential approach to the development of a multifunctional theranostic agent.

## Graphical abstract

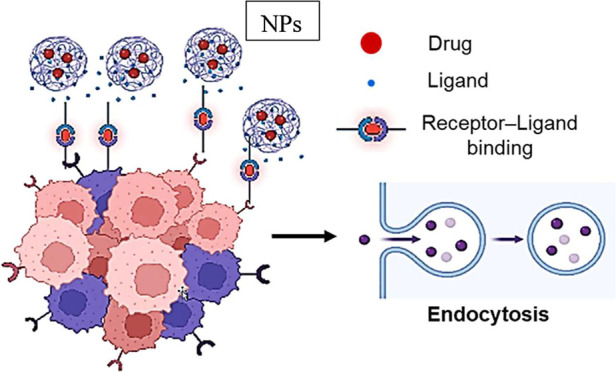

## 1 Introduction

Carbon nanodots (CNDs) are amorphous, quasi-spherical luminescent carbon nanoparticles (NPs) with various surface functional groups, providing excellent water solubility, and are easily functionalized for numerous applications.^1^ Their remarkable optical properties, such as enhanced optical fluorescence, photostability, and excitation-dependent emission, combined with their other striking features, including low toxicity, make them a potential candidate for biological applications.^2^ The carbon core and surface functional groups were attributed to their improved biocompatibility and renal clearance,^1^ where high photoluminescence, resistance to photobleaching, and rapid penetration into the nucleus of cells make them remarkable diagnostic tools for cellular bioimaging.^3^ CNDs are widely tested as bioimaging probes,^3^ antioxidants,^4–6^ anticancer agents,^7^ and nanocarriers as drug delivery vehicles to increase anticancer efficacy, including our lab work.^8^ Nevertheless, research with multifunctional CNDs for bioimaging agents and as drug vehicles for active targeting of anticancer drugs serves as a booming thrust in theranostics.

Semiconductor cobalt oxide NPs (Co_3_O_4_ NPs) have high stability in general and are multifunctional for many applications, such as sensing, magnetic resonance imaging, and biomedicine (antioxidant, anticancer, and drug delivery).^9,10^ The optoelectronic properties with their valence states in the Co_3_O_4_ NPs have made them inquisitively noteworthy for biological applications,^11^ including antibacterial,^10^ antioxidant, and anticancer activities.^12^ The Co_3_O_4_ NPs were also reported as diagnostic agents and used for targeted drug delivery by functionalizing with ligands or other small molecules or conjugating anticancer drugs.^11,13^ Albeit these applications exist, the Co_3_O_4_ NPs possess substantial toxicity in their ionic form, which is one of the challenges to be considered while using these NPs for biological applications. The synthesis approaches of these Co_3_O_4_ NPs require more energy and time-consuming techniques and are highly challenging to synthesize small-sized controlled NPs.^11^ The toxicities of metal oxide nanomaterials are reduced by combining them with carbonaceous or safer porous materials by some surface functionalization techniques, such as covering the NPs with a shell layer.^14^

Nanomaterials can be designed and tuned flexibly, immensely beneficial for developing multiple nanobio interactions within a single composite system.^15^ Core–shell NPs are used as theranostic tools for detection, imaging agents, and cancer-specific targeting.^16^ They show various advantages of introducing multifunctionalities, such as fast pharmacokinetics, improved accumulation at the target sites, and enhanced efficacy.^17^ The specific targeting of nanomaterial-based systems in comparison with conventional anticancer drugs reduces the toxicity of healthy tissues with enhanced bioavailability and improved efficiency to cancer cells passively or actively.^15,16^ To date, there is a highly critical need for designing and synthesizing multifunctional NPs for improved cancer diagnosis, imaging, and treatment strategically.

Lung cancer (LC) spreads more rapidly in humans than any other cancer, leading to early death in 85% of patients within 5 years of diagnosis; hence, it is the leading cause of cancer deaths.^18^ Histologically, LC cells are classified as nonsmall cell LCs (NSCLCs) and small cell LCs (SCLCs), among which NSCLCs account for 85% of the total LC patients. A549 cancer cells are lung adenocarcinoma cell lines, falling under a noteworthy class of NSCLCs. Passive targeting attempts to augment the nanomaterials accumulation via enhanced penetration and retention (EPR) in the tumor tissues; the active targeting process necessitates conjugating specific ligands on nanomaterials for tumor receptors.^19^ However, passive targeting is ineffective as the tumor cells usually have a leaky vasculature, where the nanomaterials leak away. As a result, the EPR effect could not be achieved effectively. The active targeting process addresses this issue by loading anticancer drugs and binding ligands to nanomaterials to provide strong affinity and specificity to tumor cells, more precisely.^15^ Generally, tumor cells have specific target molecules called receptors attached to the cell surface, while normal cells do not. The receptors on tumor cells possess a high affinity for certain specific molecules, called ligands. The tumor microenvironment contains overly expressed receptors such as EGFR (epidermal growth factor receptor), FR (folate receptor), CD44 receptors (cluster of differentiation), CD71 (transferrin), luteinizing hormone release hormone (LHRH), adenosine triphosphatases (ATPases), and chemokine receptor type 4 (CXCR4).^20^ Ligands are substances targeting these receptors with high affinity once the targeting materials containing the ligands reach the specific tumor sites.^21^ The active targeting strategy using ligand-conjugated nanomaterials to bind overexpressed receptors on the cancer cell surfaces renders site-specific delivery of drugs for tumor treatment,^15,20^ in general, by facilitating endocytosis and inhibiting the multidrug resistance (MDR) effect of tumor cell treatment.^15,20^ Specific ligands used for targeting lung cancer receptors include some small molecules such as folic acid (FA), transferrin (Trf), polymers (polyethylene glycol—PEG, polyvinylpyrrolidone—PVP, and silica (SiO_2_) NPs), and heparin (Hep).^20,21^ These molecules are conjugated with nanomaterials or anticancer drugs. Heparin and PEGylated SiO_2_–based NPs, coupled with fluorescent dyes, are ligands as well that increase the bioavailability and target the A549 cells by energy-dependent endocytosis reactions.^7,22,23^ Successful applications of the ligands to combine with the nanomaterials and anticancer drugs such as doxorubicin, curcumin, paclitaxel, and cisplatin were reported to have better targeting to the cancer cells and improved bioavailability.^21,24,25^ Cross–linking chemistry is a common strategy for coupling ligands with NPs via covalent conjugation of amidation reactions and increasing their water solubility and conjugation efficiency.^26–28^ Hence, developing a theranostic drug is essential via active targeting, multifunctional nanomaterials-based drug delivery for effective targeting using the ligands.^20,24^

Hybrid NPs can occur in various structures, such as core–shell, heterodimer, nanobranches, etc., and have been researched for multifunctional applications, such as targeted drug delivery and the development of delivery vehicles. Amid these structures, the core–shell hybrid NPs were more advantageous with inner core and outer shell materials to reveal novel properties not found in the core or shell components.^29^ Core–shell NPs with a desirable morphology and tunable pore size possess immensely appealing ascribable properties, such as multifunctional applications with improved properties; the shell material coatings can enhance biocompatibility and surface functionalization, thus reducing toxicity. Different synthesis techniques, such as physical fabrication strategies, chemical polymerization, self-assembly, sol–gel methods, and biosynthetic techniques, have been researched to synthesize biocompatible core–shell hybrid nanomaterials in a simple route for using them in biomedical applications.^29^

CNDs feature easy-to-surface functionalization and extremely small size, usually less than 10 nm. They are used as an excellent drug delivery vehicle with properties such as high fluorescence, biocompatibility, antioxidant, and high solubility for safe use in cancer diagnoses and therapy (theranostics) applications.^1,25,30^ The CND hybrid NPs would be advantageous for biological studies and impart synergetic features. In the research by Zhang et al.^31^ and Feng et al.,^32^ surface-functionalized CNDs synthesized by hydrothermal methods were shown to be used for high-resolution imaging because of their excellent photostability and stimulated emissions to the cells. Similarly, studies by Zhang et al.^31^ and Feng et al.^32^ using the hybridized and surface-functionalized CNDs showed promising anticancer and imaging potentials with specificity toward cancer cells by impairing the mitochondrial functions. The intriguing heterogeneous chemistry between the hybrid NPs, possessing interactions with van der Waals, hydrogen bonding, electrostatics, or noncovalent functionalization with π–π stacking chemistry at the interface of two or more different materials, has made researchers inquisitively approach to understanding their mechanism for multifunctional applications.^33^ The above-mentioned advantages render the multifunctionality of hybrid NPs as effective bioimaging and delivery agents.

In this work, we synthesized spherical core–shell Co_3_O_4_@CND hybrid NPs of size less than 20 nm using a simple microwave-assisted synthetic method.^8^ The Co_3_O_4_ NPs along with a carbon-based shell may provide biocompatibility in cellular delivery.^1^ This study is designed to investigate the uptake of Co_3_O_4_@CND hybrids and additional ligand bioconjugation for cancer cell targeting and their potential cytotoxic effects to yield anticancer activity. Various ligands such as folic acid, heparin, transferrin, or SiO_2_ polymers with rhodamine modification specific to A549 cancer cells were used to compare biocompatibility, targeting specificity, and cytotoxicity. The results of the Co_3_O_4_@CND hybrid NPs conjugated with an anticancer drug, DOX, increase the therapeutic effect significantly. The possible mechanism of using Co_3_O_4_@CND hybrid NPs as carriers for drug delivery and anticancer properties was discussed. Note that some content of this work is adapted from A. Jayapalan’s PhD dissertation thesis.^34^

## 2 Experimental Section

### 2.1 Materials

Chemicals, including cobalt acetate tetrahydrate (Sigma-Aldrich), pure anhydrous ethanol (Sigma-Aldrich), 25–28% ammonium hydroxide solution (Sigma-Aldrich), citric acid (ACROS Organics), ethylenediamine (EDA, Fisher Scientific), and deionized (DI) water, and dyes, such as Alamar blue (Thermo-Fisher), MitoTracker Red CMXRos (Thermo-Fisher), and dichlorofluorescein diacetate (DCFH-DA) (Sigma-Aldrich), were used in this work. Solvents, such as paraformaldehyde (Fisher Sci), phosphate buffered saline (PBS) (Thermo-Fisher), triethanolamine (Fisher Scientific), formamide (Sigma-Aldrich), anhydrous dimethyl sulfoxide (Thermo-Fisher), and ligand molecules and agents, including folic acid (FA) (Alfa Aesar), bovine serum albumin (BSA, Sigma-Aldrich), doxorubicin (DOX, Fisher Sci), heparin (Sigma-Aldrich), rhodamine (Sigma-Aldrich), polyvinylpyrrolidone (PVP, Sigma-Aldrich), polyethylene glycol (PEG, Alfa Aesar), tetraethyl orthosilicate (TEOS, Sigma-Aldrich), and transferrin (Trf, Sigma-Aldrich), were used without further purification. EAhy926 and A549 cells were purchased from ATCC. Cell culture media, Dulbecco’s modified Eagle’s medium (DMEM), and Ham’s F-12 nutrient mixture (F-12K medium) were purchased from ATCC. The fetal bovine serum (FBS, Fisher Sci), penicillin–streptomycin, Pen-Strep (Thermo-Fisher), and TrypLE buffer (Thermo-Fisher) were used for the anticancer studies in this research. All of the materials mentioned above were used in this work without further purification and are of analytical grade.

### 2.2 Material Synthesis

#### 2.2.1 Synthesis of Co_3_O_4_@CND Hybrid NPs

First, Co_3_O_4_ NPs were synthesized. Cobalt acetate tetrahydrate and absolute ethanol were used as precursors. The precursors were weighed, mixed, and stirred with ammonia by modifying a reported procedure.^35^ Then, the content was transferred to a microwave synthesizer (CEM Corp 908005 Microwave Reactor Discovery System) in a pressure-controlled, sealed environment with 300 W power, 100 psi pressure, and 150 °C temperature for 30 min. These particles were collected and purified by centrifugation at 10,000 rpm for 10 min. The washed particles were collected and dried under the furnace at 80 °C for 6 h and then labeled as Co_3_O_4_ NPs. These Co_3_O_4_ NPs were used to synthesize core–shell hybrid NPs following the CNDs’ synthesis procedure established and reported by Arvapalli et al.^8^ This modest microwave method without any organic solvents was effective in producing core–shell Co_3_O_4_@CND hybrid NPs. In addition, CNDs were synthesized to compare their characterization and biological studies. A schematic route of the synthesis of all the NPs and their conjugation strategies is shown in Figure S1.

#### 2.2.2 Co_3_O_4_@CND Hybrid NP Conjugation with FA-BSA-DOX

FA-BSA conjugation was carried out for the effective attachment of FA to the Co_3_O_4_@CND hybrid NPs by 1-ethyl-3-(3-(dimethylamino)­propyl) carbodiimide (EDC)-NHS-based cross–linking reactions using a reported procedure by Zhao et al.^36^ Then, the particles with FA and BSA were stirred for 24 h. The FA-BSA complex was collected and dialyzed with a 1 kDa membrane for 3 days to obtain purified particles and freeze-dried. Co_3_O_4_@CND hybrid NPs (5 mg in DI water mixed by sonication) and the FA-BSA complex dispersed in PBS solution (pH = 7.4) were added and stirred for 24 h again. The final brown particles were collected and dialyzed in a 1 kDa membrane for 3 days. The residues were then dried using a freeze-dryer (Labconco Free Zone 6 freeze-dryer) and labeled as FA-BSA-Co_3_O_4_@CNDs.

The DOX was loaded on FA-BSA-Co_3_O_4_@CND hybrid NPs by mixing.^36,37^ First, 2 mg of the FA-BSA-Co_3_O_4_@CND hybrid NPs was dispersed in 10 mL of PBS solution by sonicating for 1 h to disperse thoroughly. Then, 1 mg of DOX was added and stirred for 24 h in the dark. The excess of uncombined DOX was removed by centrifugation at a speed of 10,000 rpm. The precipitates were washed several times, dialyzed with a 1 kDa membrane for 24 h, and freeze-dried. The dried particles were labeled as FA-BSA-Co_3_O_4_@CNDs-DOX. The drug DOX-loading efficiency of these particles was calculated using eq 1 below by measuring the amount of doxorubicin added.^36,37^

1 $$\mathrm{DOX}\;\mathrm{loading}=\frac{\mathrm{Weight of drug DOX in NPs}}{\mathrm{Weight of the NP Carriers}}\times 100\%$$

#### 2.2.3 Co_3_O_4_@CND Hybrid NP Conjugation with Hep-DOX

Co_3_O_4_@CNDs-Hep was prepared using self-assembly and graft copolymerization techniques in a reported procedure by Zhang et al.^7^ In this procedure, heparin was grafted onto the surface of Co_3_O_4_@CNDs by amide bond formation. First, Hep (0.5 g) was first dispersed in 10 mL of formamide, and then 205 mg of EDC and 115 mg of NHS were added and stirred for 12 h in the dark. Then, the obtained colorless solution was filtered by a 0.22 μm syringe filter to remove any residues, and the solution was precipitated with acetone. After drying the precipitates at 60 °C for 24 h, a light, white, sticky solid was obtained, labeled as Hep-NHS.

The Hep-NHS (0.3 g) was dispersed in 5 mL of formamide and 5 mL of anhydrous dimethyl sulfoxide and mixed with 50 mg of Co_3_O_4_@CNDs and 1% triethanolamine. All these contents were stirred for 12 h in the dark. Then, the solution was precipitated with acetone, and the residues were collected. The collected residues, Co_3_O_4_@CNDs-Hep, were washed with ethanol to remove the unbound Hep. Finally, the Co_3_O_4_@CNDs-Hep solution was dried by freeze-drying and stored at −20 °C before use.

DOX was loaded on the Co_3_O_4_@CNDs-Hep by a dialysis method.^7^ Co_3_O_4_@CNDs-Hep (0.2 mM) was dispersed in DI water by ultrasonication at RT. DOX (0.1 mg/mL) was added to the yellow Co_3_O_4_@CNDs-Hep solution and stirred for 12 h in the dark. After thorough mixing, the solution was dialyzed (1 kDa) against DI water for 48 h. DOX drugloading capacity on Hep-Co_3_O_4_@CNDs was calculated using eq 1.

#### 2.2.4 Co_3_O_4_@CND Hybrid NP Conjugation with PVP, PEGylated SiO_2_, and Rhod

Co_3_O_4_@CND hybrid NP conjugation with PVP, PEGylated SiO_2_, and Rhod was done by modifying a reported procedure by Yoon et al. and Lu et al.^23,38^ Accordingly, a 10% aqueous ethanolic solution of PVP (50 mg of PVP in 10 mL of ethanol) was added first to the Co_3_O_4_@CND hybrid NPs to improve the chemical stability. The PVP-stabilized Co_3_O_4_@CND hybrid NPs were then separated by centrifugation at 10,000 rpm for 30 min by washing with acetone and redispersing with 10 mL of ethanol. Then, 3-aminopropyltriethoxysilane (APS) and rhodamine B (Rhod) were mixed in the dark to yield trimethoxysilane (TMS) with Rhod. A solution of TEOS and Rhod-modified TMS with a molar ratio of 0.3/0.04 was added dropwise to the ethanol solution of PVP-stabilized Co_3_O_4_@CND NPs. Ammonia solution (0.86 mL; 30 wt % by NH_3_) was injected as a catalyst in the reaction to yield NPs with PVP-Co_3_O_4_@CNDs-PEG-SiO_2_-Rhod conjugations. These particles were washed and precipitated with ethanol by centrifugation at 10,000 rpm for 50 min. The separated hybrid NPs (45 mg) were again dispersed in 10 mL of absolute ethanol and mixed with 125 mg of 2-[methoxy­(polyethyleneoxy)­propyl]­trimethoxysilane (PEG-Si (OMe)_3_; 0.02 mmol) at pH 12 (adjusted with ammonia) to improve their biocompatibility. The final particles, PVP-Co_3_O_4_@CNDs-SiO_2_–PEG-Rhod, labeled as Co_3_O_4_@CNDs-Rhod, were collected by washing and centrifuging with ethanol at 10,000 rpm for up to 60 min.

#### 2.2.5 Co_3_O_4_@CND Hybrid NP Conjugation with Trf-DOX

This conjugate was synthesized by linking the Co_3_O_4_@CNDs with EDC and NHS cross-linking reactions by covalently coupling carboxyl groups to primary amines.^39^ The stepwise procedure is described below.

First, Co_3_O_4_@CNDs (3 mg) were dispersed in 2 mL of PBS, pH 7.4, and added with EDC (6.7 mg), and the mixture was stirred at room temperature. After 30 min, a 1 mL PBS solution of 4 mg mL^–1^ NHS was added to the above solution, and the mixture was stirred for another 30 min. Then, 1 mL of 8 mg mL^–1^ PBS Trf solution was added dropwise and stirred for 2 h at room temperature. The reaction mixture was then collected and purified using a 1 kDa dialysis membrane. The conjugated Co_3_O_4_@CNDs-Trf NPs were collected and separated into two portions, where one portion was dried using a freeze-drier, and the other half was used for the DOX conjugation without further treatment.^32^

Then, in the Co_3_O_4_@CNDs-Trf conjugate solution, EDC (6.7 mg) was added and stirred for 30 min at ambient temperature. Then, NHS (4 mg) was added and stirred again for another 30 min. Then, a solution of DOX (2 mg) in DMSO (0.1 mL) and DI water (1.0 mL) was added and stirred for two more hours. The final particles were purified by dialyzing using a 1 kDa membrane for 3 days. Co_3_O_4_@CNDs-Trf-DOX conjugates were then freeze-dried and stored at −20 °C for further characterization and cell viability studies.^39^ DOX drug-loading capacity was calculated for Co_3_O_4_@CNDs-Trf-DOX by using eq 1.

### 2.3 Materials Characterization

The morphologies of the Co_3_O_4_@CND hybrid NPs and all other synthesized NPs, such as FA-BSA-Co_3_O_4_@CNDs-DOX, Hep-Co_3_O_4_@CND-DOX, Co_3_O_4_@CND-Rhod, and Co_3_O_4_@CND-Trf-DOX hybrids, were characterized and compared using transmission electron microscopy (TEM, Carl Zeiss Libra 120 Plus). The hybrid NPs’ properties were compared with CNDs and Co_3_O_4_ NPs in further characterizations.

In addition, the ligand-conjugated synthesized NPs were characterized to understand their changes in the structures and optoelectronic properties before and after conjugation. The comparison of these synthesized NPs was performed with their counterparts, including FA, FA-BSA, FA-BSA-Co_3_O_4_@CNDs, Hep-NHS, Hep-Co_3_O_4_@CNDs, Trf, Trf-Co_3_O_4_@CNDs, DOX, and Co_3_O_4_@CNDs. The characterization studies, such as ultraviolet (UV)–visible absorbance (Agilent), photoluminescence (PL) spectroscopy (Horiba Spectrophotometer), Fourier transform infrared (FTIR) spectroscopies (Varian 670), and Malvern Zetasizer dynamic light scattering (DLS, Malvern Instruments ZEN3600), were performed for each of the synthesized NPs and their respective individual counterparts for evaluating their optical, structural, and surface charge properties for using them as an effective anticancer agent.

### 2.4 Cellular Studies

#### 2.4.1 Cell Culture

EAhy926 endothelial and A549 adenocarcinomic lung epithelial cell lines were cultured in DMEM and F-12K medium, containing 10% fetal bovine serum (FBS) and 1% Pen-Strep, and are grown in a CO_2_ incubator (5%) at 37 °C. After being cultured and grown, these cells are passaged with TrypLE/EDTA and cultured for further biological assays.

#### 2.4.2 Cellular Uptake and Subcellular Localization Analysis of Co_3_O_4_@CND Hybrid NPs

The cellular uptake and subcellular localization of the Co_3_O_4_@CND hybrid NPs were studied and compared by using confocal microscopy. First, 1 × 10^5^ cells were seeded on coverslips and positioned in 12-well plates. EAhy926 and A549 cells were treated with Co_3_O_4_@CND hybrid NPs and CNDs at concentrations of 0, 0.4, and 0.8 mg/mL in designated wells and were analyzed in triplicate, respectively. After 20–24 h of treatment, the cells were fixed with paraformaldehyde and stained with MitoTracker Red CMXRos dye (0.2 μM for EAhy926 and 0.1 μM for A549 cells, 10 min, 37 °C, Molecular Probes, λ_ex_/λ_em_ at 579/599 nm)^8,40^ to stain the actin filaments in mitochondria. Cells were washed with PBS twice before imaging. Co_3_O_4_@CND hybrid NPs and CNDs with concentrations of 0, 0.4, and 0.8 mg/mL were imaged at oil immersion of 63× using a confocal microscope to confirm their viability and uptake in EAhy926 and A549 cells. The subcellular localization of dye and NPs was observed by simultaneously imaging cells on coverslips for the Co_3_O_4_@CND hybrid NPs (with a 0.4 mg/mL concentration). Imaging was performed under a Zeiss Z1 spinning disk confocal microscope using a greater magnification, 100× oil immersion objective lens, for a deeper understanding of subcellular localization.^40,41^ The NPs and MitoTracker Red concentrations were optimized to exclude interference,^40,41^ using the Rhod channel for mitochondrial layers in cells and 4′,6-diamidino-2-phenylindole (DAPI) channels for hybrid NPs. Co_3_O_4_ NPs did not present fluorescence in cells and were not used for comparison in this study.

#### 2.4.3 Intracellular Antioxidant Measurements

The DCFH-DA assay monitors the intracellular reactive oxygen species (ROS) levels in EAhy 926 and A549 cells, where the DCFH-DA acts as an oxidative stress and hydrogen peroxide (H_2_O_2_) probe.^42^ Intracellular ROS, including H_2_O_2_, hydroxyl radical (OH^–^), and superoxide anion (O_2_^–^), contribute to the major physiological processes inside normal human cells and cancers. First, 1 × 10^4^ cells were seeded in a 96-well plate and cultured for 24 h. Then, the cells were treated with different concentrations of Co_3_O_4_@CND hybrid NPs, Co_3_O_4_ NPs, and CNDs for 24 h, such as 0–0.8 mg/mL. The cells were washed twice with 1X PBS, and 10 and 20 μM of DCFH-DA probe in FBS-free media were added to the treated EAhy926 and A549 cells, respectively. After incubating for 30 min at 37 °C, the cells were washed twice with 1× PBS to remove the dye interference and then replaced with 1× PBS for the measurement in the plate reader. The cells with PBS were incubated at 37 °C for 5 min, and the fluorescence intensity was measured at λ of excitation of 485 and emission of 530 nm. These measurements measure the oxidation of DCFH-DA to “2′,7′-dichlorofluorescein” (DCF) by intracellular ROS generation. As controls, the cells without NP treatment and the NP-treated cells with no DCFH-DA are used. Cells treated with ascorbic acid (AA), a powerful antioxidant, were compared as a negative control. The normalized fluorescence intensity from the plate reader measurements was calculated by subtracting the blank (no dye-treated cells).

#### 2.4.4 Biocompatibility and Cytotoxicity Studies

A viability assay was performed using the Alamar Blue test. Percentage viability was carried out for the Co_3_O_4_@CND hybrid NPs and compared with their counterparts, Co_3_O_4_ NPs and CNDs, in EAhy926 and A549 cells. Briefly, 1 × 10^4^ cells were seeded in every well in a 96-well plate with the DMEM and F12K complete media and incubated for 24 h. Then, the cells were treated with varying concentrations ranging from 0 to 0.8 mg/mL of Co_3_O_4_@CND hybrid NPs, Co_3_O_4_ NPs, and CNDs for 24 h. As a control, the cells without NPs and varying concentrations of NPs without cells were used to study the extent of cytotoxicity in the cells. The Alamar Blue assay quantitatively determines cell viability, which is evaluated by the metabolic reactions. When Alamar Blue was added to the cells, its oxidized form penetrated via the cytosol, thereby reducing the mitochondrial activity by accepting electrons from enzymes such as NADPH, FADH, FMNH, and NADH, as well as from the cytochromes.^43^ The measurements were read on a plate reader at wavelengths of excitation of 560 nm and emission of 590 nm, representing the number of living cells. The percentage of viable cells was calculated using eq 2.

2 $$viability\;\left(\%\right)=\frac{Fl\;\left(treatment\right)-Fl\;\left(blank\right)}{Fl\;\left(control\right)-Fl\;\left(blank\right)}$$

where Fl is the fluorescence intensity. The percentage viability of all the synthesized NPs of Co_3_O_4_@CND hybrids with ligand and DOX conjugation was determined using the same AB assay protocol by comparing them with the respective ligands, DOX, and Co_3_O_4_@CND hybrid NPs without conjugation to compare their biocompatibility and cytotoxicity in EAhy926 and A549 cells, respectively. In addition, the counterparts, such as FA, FA-BSA, FA-BSA-Co_3_O_4_@CNDs, Hep-NHS, Hep-Co_3_O_4_@CNDs, Trf, Trf-Co_3_O_4_@CNDs, DOX, and Co_3_O_4_@CNDs, were compared in the viability studies in EAhy926 and A549 cells to understand the anticancer activity of the conjugated ligands. The effective drug dosage, IC_50_, was 4 mg/mL in a reference stated, and a maximum concentration of 0.4 mg/mL (1/10th of IC_50_) was chosen in their biological studies.^22^ Similarly, in our study, we used increased concentrations of Co_3_O_4_@CND hybrid NPs conjugated with the ligands, such as 0, 0.1, 0.2, and 0.4 mg/mL, to measure their extent of biocompatibility and cancer-targeting effect safely.

### 2.5 Data Analysis

Each assay was carried out with three independent experiments. The confocal microscopic images were analyzed using AxioVision 4.8 and ImageJ software. The mean and standard error (SE) were calculated, and the data and their respective significant differences were analyzed using Microsoft Excel. An asterisk was indicated for significance at a probability of *P* < 0.05 compared to the 0 mg/mL from a one-tailed *t*-test analysis.^40^ The fluorescence intensities in the quantified histograms in each plate reader measurement were subtracted from the background (blank) fluorescence.

## 3 Results and Discussion

### 3.1 Physicochemical Properties and Characterization of Synthesized NPs

The synthesized NPs were characterized by using microscopy and spectroscopy tools for morphological, structural, and property analyses. The TEM images of the dried Co_3_O_4_@CNDs before and after conjugation with NPs are shown in Figure 1a–e. The synthesized Co_3_O_4_@CND NPs (Figure 1a) were spherical and uniform. The ImageJ analysis of the TEM image showed that the average size of the core–shell structures was 14.7 ± 3.7 nm, with a core diameter of 11.9 ± 2.9 nm and a shell thickness of 2.8 ± 0.4 nm surrounding the core structure. The TEM images showed that ligand-conjugated NPs, FA-BSA-Co_3_O_4_@CNDs-DOX, Hep-NHS-Co_3_O_4_@CNDs-DOX, Co_3_O_4_@CNDs-Rhod, and Co_3_O_4_@CNDs-Trf-DOX (Figures 1b–e), had their average sizes in the range of 19.0 ± 3.0, 29.5 ± 2.5, 34.0 ± 2.0, and 30.0 ± 6.0 nm, respectively. The TEM images of Co_3_O_4_@CNDs-Rhod (Figure 1d) and Co_3_O_4_@CNDs-Trf-DOX (Figure 1e) showed shell layers, which were expected because of the CND covering, PEGylation, and the conjugation of Trf with DOX, increasing the size of Co_3_O_4_@CNDs.

**Figure 1:**
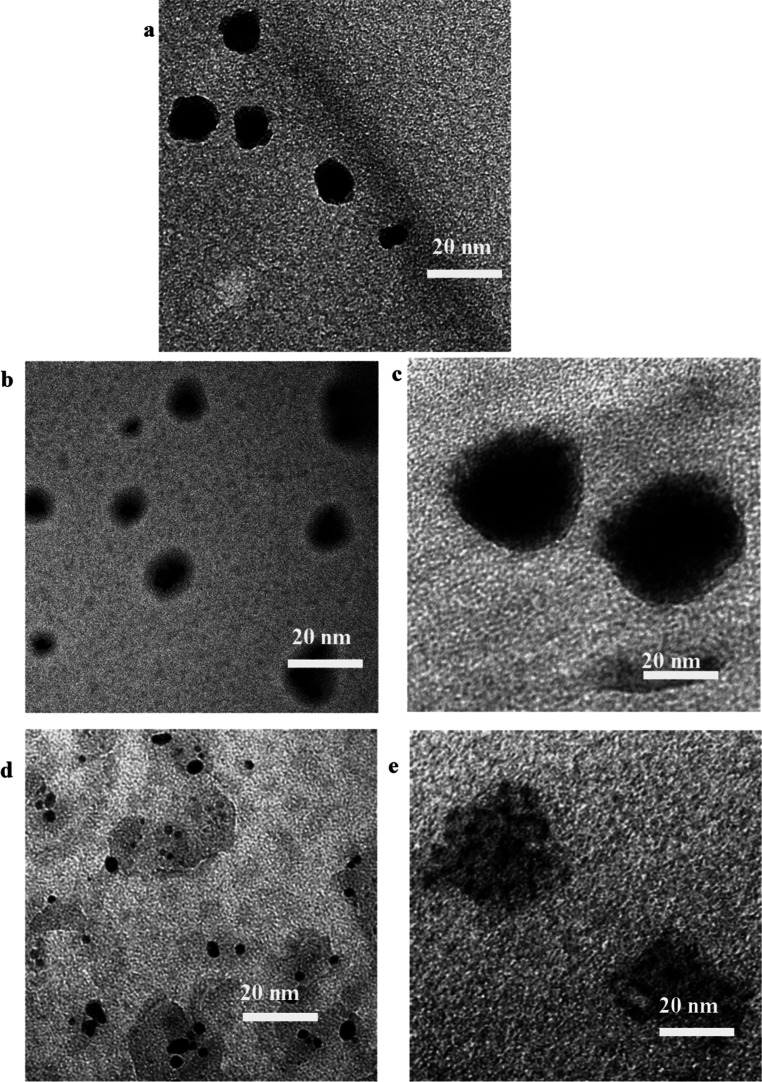
TEM images of (a) Co_3_O_4_@CNDs, (b) FA-BSA-Co_3_O_4_@CND-DOX, (c) Hep-Co_3_O_4_@CND-DOX, (d) Co_3_O_4_@CND-Rhod, and (e) Co_3_O_4_@CND-Trf-DOX hybrids.

Using eq 1, the DOX-loading efficiencies (the ratio to the hybridized NPs) of FA-BSA-Co_3_O_4_@CNDs, Hep-Co_3_O_4_@CNDs, and Co_3_O_4_@CNDs-Trf were calculated as 96.25%, 93.75%, and 99.7% (wt), respectively. It is expected that their size and ligand modifications were advantageous for selectively delivering Co_3_O_4_@CNDs and DOX into A549 cells with FA receptors.^20,24^

The FTIR spectra of the four different synthesized NPs before and after conjugation were compared to those of DOX (Figure 2). The FTIR spectra demonstrated that the synthesized NPs with FA-BSA-Co_3_O_4_@CND-DOX, Hep-Co_3_O_4_@CND-DOX, Co_3_O_4_@CND-Rhod, and Co_3_O_4_@CND-Trf-DOX hybrids exhibited the characteristic ligand peaks of FA, Hep, Rhod, Trf, and DOX, respectively, confirming the respective attachments. The FTIR spectra of the conjugates were individually compared with their counterparts, such as FA, FA-BSA, and FA-BSA-Co_3_O_4_@CNDs for the FA-BSA-Co_3_O_4_@CNDs-DOX conjugates (Figure S2a), Hep and Hep-Co_3_O_4_@CNDs (Figure S2b), Sulfo-Rhod (Figure S2c), and Trf-Co_3_O_4_@CNDs and DOX (Figure S2d). The FTIR spectra of the Co_3_O_4_@CND hybrid NPs showed peaks corresponding to Co (II) and Co (III) at 578 and 665 cm^–1^, the valence states of Co_3_O_4_, as well as −C–C– (1542 cm^–1^), –C=N (1635 cm^–1^), –CH (2900–3050 cm^–1^), and –OH (3100–3400 cm^–1^) bonds, representing characteristics of CNDs.^44–46^ In contrast, after conjugation, the FTIR spectra (Figure S2a) of FA-BSA-Co_3_O_4_@CNDs and FA-BSA-Co_3_O_4_@CNDs-DOX showed the typical stretching vibration peak at 937 cm^–1^, attributed to FA.^47^ Additional peaks for C=N double bond (1640 cm^–1^), −N–H (1564 cm^–1^), amide (1700 cm^–1^) bonds, and partial benzene ring vibrations (1351 cm^–1^), which are characteristics of DOX,^48^ were observed in FA-BSA-Co_3_O_4_@CNDs-DOX, confirming the attachment of DOX. The FTIR (Figure S2b) comparison of Hep-NHS-Co_3_O_4_@CNDs with its respective DOX modification demonstrated the presence of functional groups, such as Hep (−COO^–^ functionalization at 1612 cm^–1^) and NHS (ester group at 1693 cm^–1^, formed during conjugation of Hep and NHS), and the peak representing the DOX structure (1643 cm^–1^).^7,49^ The FTIR (Figure S2c) comparison of Co_3_O_4_@CNDs-Rhod and Sulfo-Rhod with Co_3_O_4_@CNDs showed many new peaks corresponding to PEG, Rhod, and SiO_2_ functionalization. The symmetric and asymmetric stretching vibration peaks of C–O–C were located at 1034 and 1234 cm^–1^, respectively, indicating that PEG-Co_3_O_4_@CNDs functionalization possessed hydroxyl, carbonyl, and carboxylic groups. The strong peak at 1542 cm^–1^ was characteristic of the stretching of −N–O bonds, whereas the peaks at 1600 to 1300 and 2942 cm^–1^ represented graphitic bonds and –CH_2_ from PEG.^50,51^ The smaller peaks at 1042 and 732 cm^–1^ were due to SiO_2_ functionalization (−Si–O–C and −Si–O–Si– symmetrical stretching vibration shift) present on the particle surfaces because of TEOS condensation with carbon in the conjugates.^52^ In the comparison spectra, the Co_3_O_4_@CND peaks in FA-BSA-Co_3_O_4_@CNDs-DOX and Co_3_O_4_@CNDs-Rhod appeared weakened because of the structures of the polymeric outer shell coverings, such as FA, BSA, PEG, SiO_2_, or Sulfo-Rhod. The FTIR (Figure S2d) comparison of Co_3_O_4_@CNDs-Trf suggested conjugation by the stretching vibration peaks, such as –C=O in the amido (II) bond (1634 cm^–1^), being formed in EDC-NHS chemistry. In addition, the benzene ring (1410 cm^–1^) in the DOX structure was observed in Co_3_O_4_@CNDs-Trf-DOX.^26^

**Figure 2:**
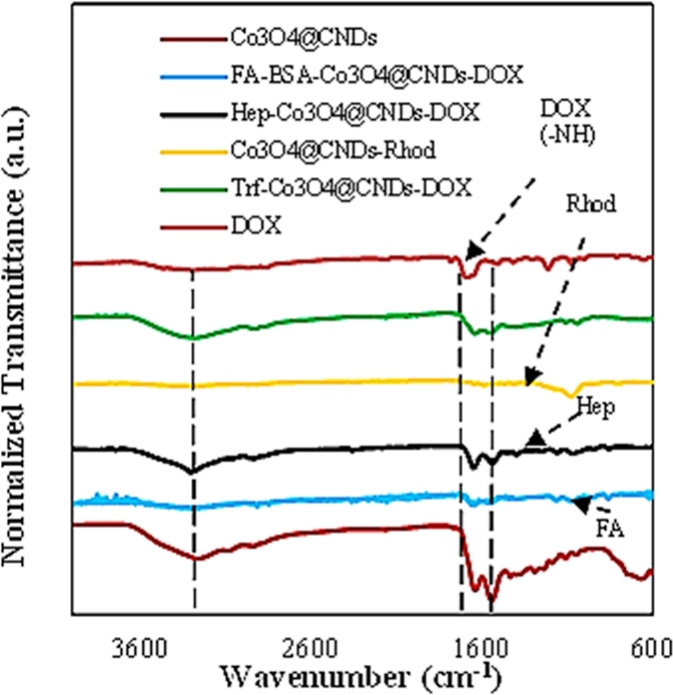
Comparison of FTIR spectra of Co_3_O_4_@CND hybrid NPs, FA-BSA-Co_3_O_4_@CND-DOX, Hep-Co_3_O_4_@CND-DOX, Co_3_O_4_@CND-Rhod, Co_3_O_4_@CND-Trf-DOX hybrids, and DOX.

The optical photoelectronic properties of NPs, such as absorbance and fluorescence, play an important role in bioimaging in human cells, viability measurements, and other biological studies.^53,54^ UV–visible and PL spectroscopies (Figure 3a,b) were used to measure the absorbance and fluorescence of the Co_3_O_4_@CND hybrid NPs and the four mentioned conjugates compared with DOX. The absorbance band of the Co_3_O_4_@CND hybrid NPs at 254 nm in the UV–visible spectrum was attributed to the π–π* transition of C=C bonds in sp^2^ domains.^8^ The band at 360 nm of the Co_3_O_4_@CND hybrid NPs was characteristic of *n*–π intramolecular transitions of the –C=O surface states.^40,55^ The PL spectroscopy data showed that when excited at 360 nm, the Co_3_O_4_@CND hybrid NPs emitted strongly at 450 nm with maximum fluorescence, which was consistent with the CNDs.^40^ The UV–visible and PL spectroscopic characterizations of the synthesized NPs with ligand and DOX conjugations (Figure 3a,b) showed noticeable shifts in their absorbance and PL maximum intensities, confirming that the ligand conjugations were successful. The absorbance and fluorescence were measured with different Co_3_O_4_@CND hybrid NP concentrations using UV–visible and PL spectroscopies to calculate the quantum yield (QY). The QY of the NPs was calculated based on the above characterization results using normalized absorbance and fluorescence according to eq 3.^49,51^

3 $$\phi_{C}=\phi_{QS}\times {\left[\frac{I_{C}}{I_{QS}}\right]}^{2}\times {\left[\frac{\eta_{C}}{\eta_{QS}}\right]}^{2}$$

where ϕ, I, and η are QY, intensity, and refractive index of water (1.33), respectively, with subscripts C and QS representing the Co_3_O_4_@CND hybrid NPs and quinine sulfate, respectively. In addition, the integrated fluorescence intensity was calculated using the cumulative absorbance and fluorescence intensities, where ϕ_QS_ = 0.54 and (η_c_^2^/η_QS_^2^) = 1. Using this formula, the QY measured for the Co_3_O_4_@CND hybrid NPs was 49.63 ± 1.3%, which was close to that of the CNDs (53.2 ± 0.6%). The reduced QY of the Co_3_O_4_@CND hybrid NPs compared to CNDs was attributed to their hybridization with Co_3_O_4_ NPs, which might have slightly decreased the QY because of the chemical nature of the hybrid bond formation and the abundance of surface energy in hybrid NPs.^49^

**Figure 3:**
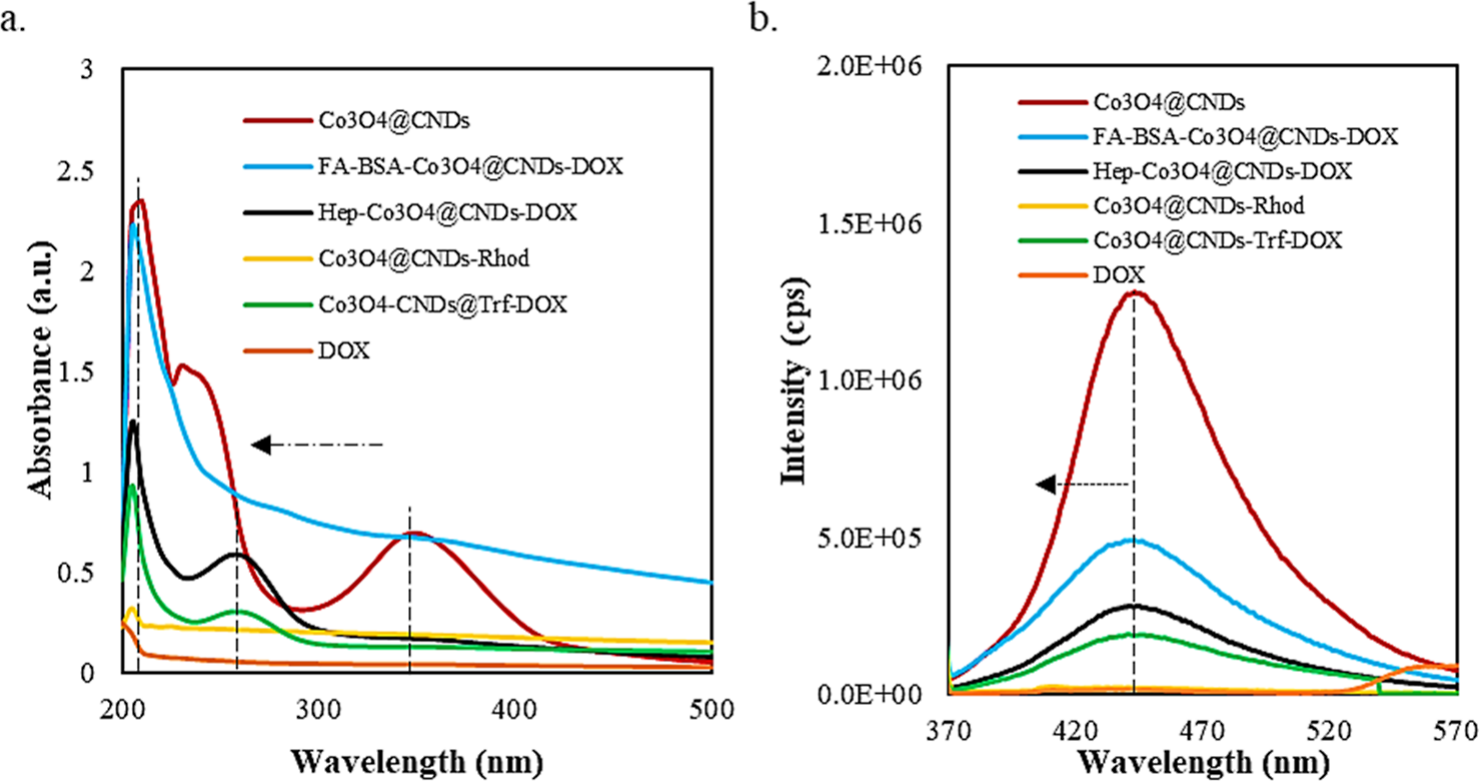
Comparison of (a) UV–visible absorbance and (b) PL spectra of Co_3_O_4_@CNDs, FA-BSA-Co_3_O_4_@CND-DOX, Hep-Co_3_O_4_@CND-DOX, Co_3_O_4_@CND-Rhod, Co_3_O_4_@CND-Trf-DOX hybrids, and DOX.

The UV–visible absorbance spectra of the conjugates were individually compared with their respective counterparts, such as FA, FA-BSA, and FA-BSA-Co_3_O_4_@CNDs for the FA-BSA-Co_3_O_4_@CNDs-DOX conjugates (Figure S3a), Hep and Hep-Co_3_O_4_@CNDs (Figure S3b), Sulfo-Rhod (Figure S3c), and Trf-Co_3_O_4_@CNDs and DOX (Figure S3d). In the UV–visible absorption spectra of FA-BSA-Co_3_O_4_@CNDs compared with those of DOX (Figure S3a), the main absorbance peak of Co_3_O_4_@CNDs at 350 nm was slightly shifted after attaching FA-BSA and DOX. The absorbance peak of FA-BSA-Co_3_O_4_@CNDs with DOX was tailing around 229 nm. There was an increase in absorbance intensity in the same tailing absorbance peak as in FA-BSA-Co_3_O_4_@CNDs, confirming the effective loading of DOX. The increase in absorbance intensities after DOX conjugation was caused by the strong hydrogen bonds and π–π interactions between them.^36,56^ After conjugating Co_3_O_4_@CNDs with Hep-NHS, the absorbance peak shifted from 350 to 260 nm, which was characteristic of Hep-NHS,^7^ and the absorbance intensity at 260 nm increased compared to Hep-NHS (Figure S3b). The UV–visible absorption spectra (Figure S3c) and PL spectroscopy (Figure S4c) confirmed complete conjugation with Rhod because the Co_3_O_4_@CNDs-Rhod intensity decreased compared to that of Co_3_O_4_@CNDs. The conjugates showed little absorbance or fluorescence intensity because of the PEGylation effect, which might have completely covered the Co_3_O_4_@CNDs-Rhod NPs. Photobleaching might have been an additional factor in decreasing the absorbance intensities of the Rhod dye complex.^23^ The absorbance maximum peak of Trf at 279 nm shifted slightly after conjugation with Co_3_O_4_@CNDs (Figure S3d). In Co_3_O_4_@CNDs-Trf-DOX, the absorbance maximum hypsochromically shifted from 279 to 260 nm, which was expected mainly because of DOX conjugation.^53^ An increase in the absorbance intensity was observed after DOX modification in Co_3_O_4_@CNDs-Trf-DOX compared with Co_3_O_4_@CNDs-Trf.

Similarly, the PL spectra with counterparts of synthesized conjugates are shown in Figure S4a–d. The PL spectroscopy of the FA-BSA-Co_3_O_4_@CNDs (Figure S4a) showed that the intensity of the Co_3_O_4_@CNDs at 440 nm largely decreased after modification with FA-BSA. In contrast, after modification with the DOX, the intensity dropped considerably. This substantial drop in the fluorescence intensity of the conjugates was expected because of DOX, which largely caused π–π stacking of molecules.^56^ The PL intensity of Co_3_O_4_@CNDs was largely quenched after modification with Hep-NHS to a greater extent, and the emission shifted slightly from 453 to 443 nm (Figure S4b). The Rhod-conjugated hybrid PL is almost quenched (Figure S4c). The Hep-NHS intensity at 440 nm was negligible. The Co_3_O_4_@CNDs intensity largely decreased after modification with Trf (Figure S4d). After modification with Trf and DOX, the intensity was still quenched with a hypsochromic shifting of the emission spectrum from 448 → 442 to 420 nm, thereby confirming effective conjugation.

The excitation dependency (ED) plots of the PL emission spectra are important features that contribute to bioimaging and biotherapeutic applications.^51^ The ED plots of the NPs from the PL spectra are shown in Figure S5a–e. The ED plots of the CNDs (Figure S5a) and Co_3_O_4_@CND hybrid NPs (Figure S5b) were analyzed at different wavelengths. The CNDs and Co_3_O_4_@CND hybrid NPs were excitation-dependent at 360 nm. The CNDs and Co_3_O_4_@CND hybrid NPs showed similar excitation wavelength dependences until 380 nm. However, after an excitation of 400 nm, they shifted considerably with lower emission intensities at different wavelengths in both spectra. From the PL spectroscopic data, we observed that the conjugated ligands possessed different emission maximum wavelengths. The ED plots of FA-BSA-Co_3_O_4_@CNDs-DOX (Figure S5c) at various wavelengths showed different emissions, indicating that they were excitation-independent. The ED plot (Figure S5d) of Hep-NHS-Co_3_O_4_@CNDs-DOX, excited at various wavelengths until 360 nm, showed emissions at the same wavelength with excitation-dependent fluorescent behavior, whereas when excited at wavelengths greater than 380 nm, the emission maximum shifted to the red region. At each excitation wavelength, two characteristic emission peaks were observed at 442 nm, representative of Hep-NHS, and a split peak at 536 and 593 nm, characteristic of DOX^7^ interactions with many proteins, such as growth factors and chemokines. The PL plot of Co_3_O_4_@CNDs-Trf-DOX (Figure S5e) showed that these NPs were excitation-independent. These excitation–dependent and –independent emission spectra are beneficial for fluorescent properties in biological applications.^52,53^

The zeta (ζ) potentials of the NPs, ligands, and DOX are shown in Figures S6a,e, and the results are compared in Table S1. The ζ potential provides information about the surface charge and dispersion stability of the NPs, which is essential in biological studies to deliver to the cells effectively.^49^ The ζ potential of the Co_3_O_4_@CND hybrid NPs was −4.3 mV. After modification with FA-BSA, the negative charge of the ζ potential (Figure S6a) of FA-BSA-Co_3_O_4_@CNDs increased, making the structure more stable with monodispersibility. However, after DOX conjugation, the negative charge of Co_3_O_4_@CNDs increased from −19.1 to −11.8 mV, demonstrating conjugation. FA possessed very few functional groups for bioconjugation; hence, modification with BSA increased the solubility of the NP conjugates.^37^ FA-BSA conjugates, which are typically used as carriers to conjugate drugs (i.e., DOX), are advantageous for selective and sensitive attachment to cancer cells and increasing the biological applicability of composites.^47^ In the Hep-NHS-conjugated Co_3_O_4_@CNDs, the negatively charged ζ potential (Figure S6b) of Hep-NHS-Co_3_O_4_@CNDs increased, making the structure more stable because of the sulfate and carboxylate groups in the –NHS groups. The high negative charge of Hep mediated the electrostatic interactions with many proteins such as growth factors and chemokines. However, after DOX conjugation, the negative charge decreased, suggesting its conjugation with Hep-NHS-Co_3_O_4_@CNDs via electrostatic interactions.^7^ Co_3_O_4_@CNDs-Rhod showed an increase in the negative charge of the ζ potential (Figure S6c), as the structure became more stable with PEG and SiO_2_ linkages. After Co_3_O_4_@CNDs modification with Trf, an increase in the negative charge of the ζ potential (Figure S6d) of Co_3_O_4_@CNDs-Trf-DOX was observed; the structure was more stable because of the Trf isoelectric point of 5.6 and the negative charge under neutral conditions.^54^ DOX was positively charged because of π–π stacking and electrostatic interactions between the Co_3_O_4_@CNDs-Trf and DOX. The negative charge of the Co_3_O_4_@CNDs-Trf-DOX increased, confirming the loading of DOX.^26^

### 3.2 Cellular Studies

#### 3.2.1 Bioimaging Studies of Co_3_O_4_@CND Hybrid NPs

The imaging of cellular uptake of the Co_3_O_4_@CND hybrid NPs in cell lines EAhy926 and A549 at various concentrations (0, 0.4, and 0.8 mg/mL) was performed by using confocal microscopy. The red-stained region represented the mitochondrial layer of cells stained with MitoTracker Red dye, whereas blue fluorescence was observed from the NPs. The merged images of the cells showed the NP uptake in the mitochondrial and nuclear regions.^40^ The Co_3_O_4_ NPs were not used for comparison because they were not fluorescent in cells to be imaged by using confocal microscopy. Cells without NPs (0 mg/mL) were used as a control for comparison while imaging the cellular uptake at increasing concentrations. Hence, no fluorescence was observed in the DAPI channel. At a Co_3_O_4_@CND hybrid NP concentration of 0.4 mg/mL, the imaged cells were significantly more viable than those at 0.8 mg/mL in the cell lines. At a Co_3_O_4_@CND hybrid NP concentration of 0.8 mg/mL, A549 cells showed viability significantly lower than that of EAhy926 cells. These cellular uptakes of Co_3_O_4_@CND hybrid NPs demonstrated their viability and uptake by cells. Furthermore, the fluorescence intensity increased with an increase in the Co_3_O_4_@CND hybrid NP concentration.

Figure 4 compares the Co_3_O_4_@CND hybrid NPs at a concentration of 0.4 mg/mL in the EAhy926 and A549 cells at 100X. The Co_3_O_4_@CND hybrid NPs displayed brighter blue fluorescence in cells because of their high absorption and QY, approximately 50%, generating considerably greater fluorescence than small organic fluorophores from synergetic bond formation and CNDs.^55,57^ It shows that the subcellular localization of the Co_3_O_4_@CND hybrid NPs in the mitochondria and nucleus increased significantly at higher concentrations (Figures S7 and S8). This is represented by the bright blue fluorescence in A549 and EAhy26 cells in the merged images. Hence, we confirmed that the Co_3_O_4_@CND hybrid NPs were taken up by both cell lines, demonstrating their application in bioimaging studies.

**Figure 4:**
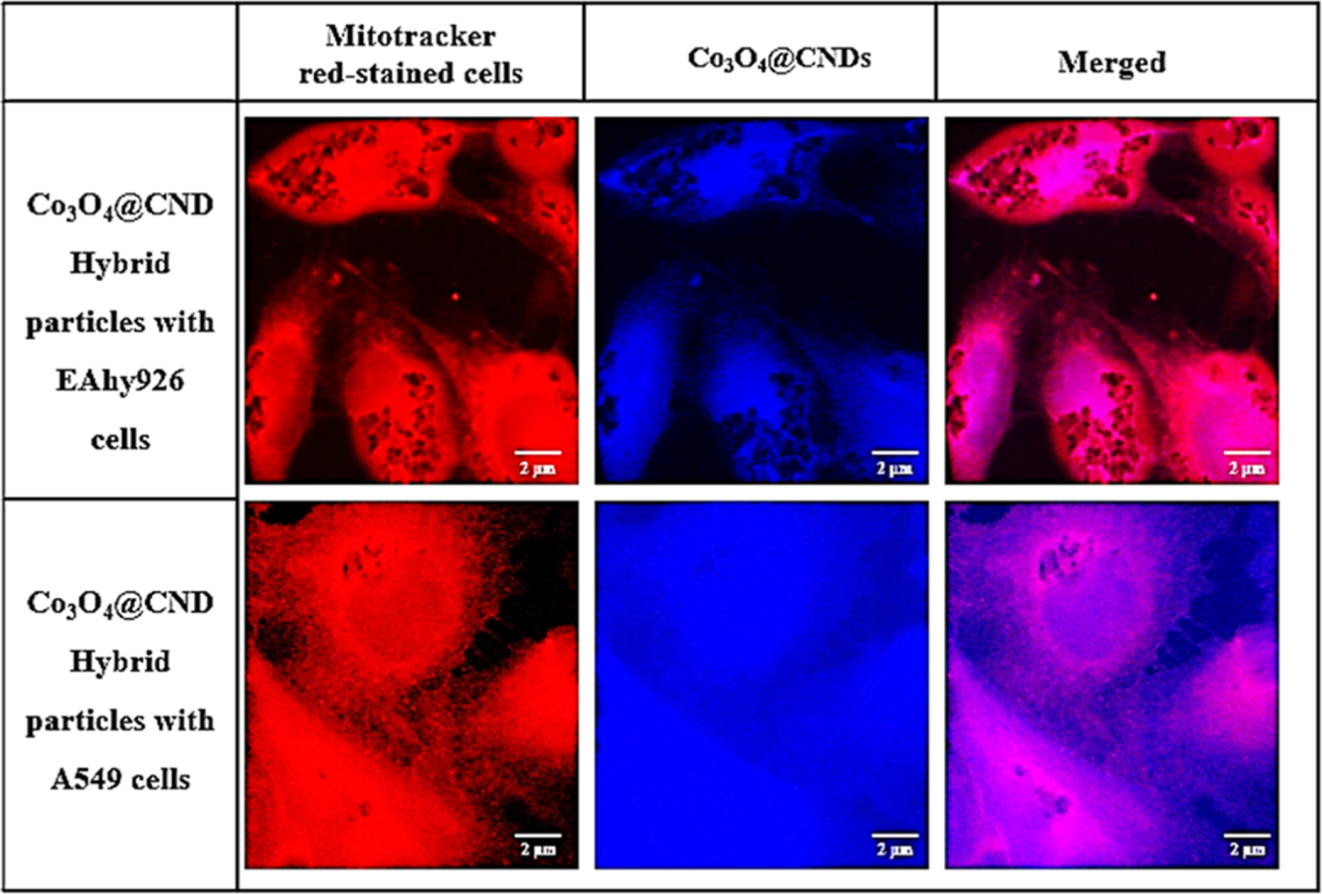
Subcellular localization of Co_3_O_4_@CND hybrid NPs in EAhy926 and A549 cells at 100× magnification.

Confocal microscopy images were further analyzed to compare the subcellular localizations of the Co_3_O_4_@CND hybrid NPs (Figure 4) and CNDs (Figures S7–S9). The magnification at 100× provided a better visual understanding of the uptake of the Co_3_O_4_@CND hybrid NPs in both normal human and cancer cells, with fluorescence observed around the mitochondrial layers and nucleus, indicating that these NPs could be used as bioimaging agents in normal and cancer cells.^36^ The nucleus penetration may aid in advanced anticancer targeting.^41^ Figure S9 indicates that Co_3_O_4_@CND hybrid NPs were more effective at reaching and targeting the nucleus than CNDs in the EAhy926 and A549 cell lines. The Co_3_O_4_@CND hybrid NPs showed better fluorescence (bright solid blue) because of their increased absorption.^57^

To analyze the subcellular localization of the Co_3_O_4_@CND hybrid NPs more quantitatively, we used Pearson’s correlation coefficient (*r*) by measuring the linear correlation between the MitoTracker Red localization and the Co_3_O_4_@CND hybrid NPs in cells using ImageJ software.^8,40^ The subcellular localization of Co_3_O_4_@CND hybrid NPs was first measured by the fluorescence intensity plot profiles using ImageJ, which showed that the red and blue channel intensities overlap in particular regions of interest from the images of Figure 4. The overlap of fluorescence intensity plot profiles in both cells is shown in Figure S10 for a clear understanding. Then, the coefficient values, *r*, for the uptake of the Co_3_O_4_@CND hybrid NPs were calculated as 0.74 and 0.85 in EAhy926 and A549 cells, respectively. The Pearson coefficient values confirmed the correlation between MitoTracker Red and the extent of localization of Co_3_O_4_@CND hybrid NPs at the mitochondrial targeting in both cells.

#### 3.2.2 Antioxidation Studies of Co_3_O_4_@CND Hybrid NPs

Intracellular enzymes in cells cleave the ester bonds of DCFH-DA dye, resulting in nonfluorescent intermediates that oxidize further to produce the highly fluorescent product, DCF.^5^ The release of fluorescent DCF in cells can be measured at 528 nm by exciting at 485 nm.^5,58^ The detected fluorescence intensity represented intracellular ROS levels.^40^ Figure 5a,b illustrates the changes in the ROS levels of the EAhy926 and A549 cells treated with Co_3_O_4_@CND hybrid NPs, CNDs, and Co_3_O_4_ NPs, compared to AA. A decrease in the fluorescence intensity was observed in the Co_3_O_4_@CND hybrid NP, Co_3_O_4_ NP, CND, and AA-treated EAhy926 and A549 cells in a concentration-dependent pattern. AA, an antioxidant and ROS inhibitor, was used as a control in this study.^5^ In A549 cells, AA treatment produced comparable results to CND treatment with increasing concentrations but with less ROS level. Compared with the CNDs and Co_3_O_4_ NPs, the ROS levels decreased rapidly in cells with Co_3_O_4_@CND hybrid NPs. At a concentration of 0.8 mg/mL, the Co_3_O_4_ NPs exhibited higher fluorescence than the AA treatment, indicating the presence of higher ROS levels.^59^

**Figure 5:**
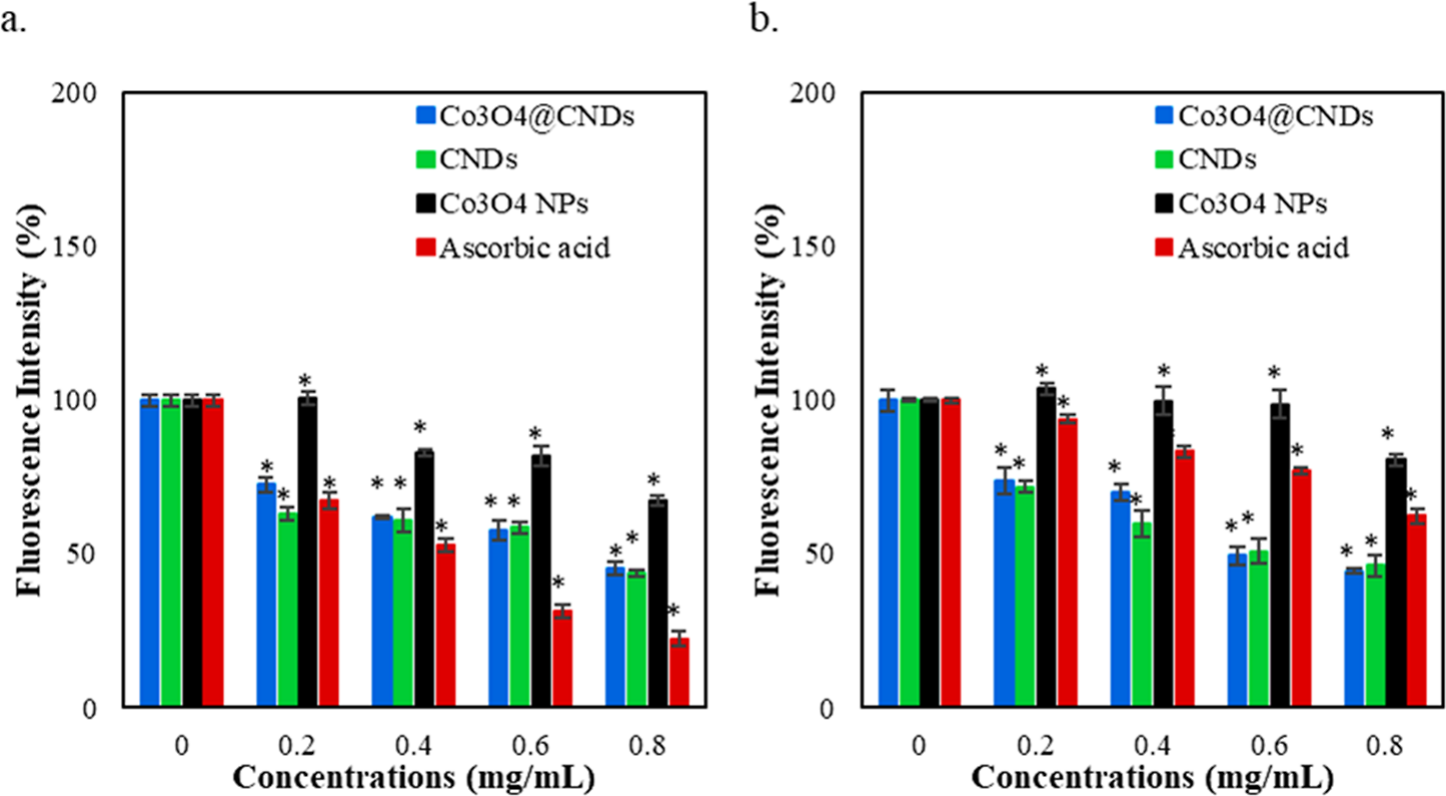
DCFH-DA assay results of Co_3_O_4_@CNDs, CNDs, Co_3_O_4_ NPs, and AA in (a) EAhy926 and (b) A549 cells. Values were obtained as the mean ± SE from three independent experiments. Each treatment was performed independently. * represents *p* < 0.05 versus control (0 mg/mL).

Figure S11 compares the antioxidative results of the Co_3_O_4_@CND hybrid NPs in EAhy926 and A549 cells. The ROS level in both cell lines decreases along with the increase of the concentration of the hybrid NP treatment. High concentrations (>0.6 mg/mL) of the Co_3_O_4_@CND hybrid NPs in the cell lines greatly reduced ROS (60%), demonstrating their role in the intracellular antioxidant effect.^5,58^ Thus, we infer that the Co_3_O_4_@CND hybrid NPs could cause significantly less ROS level in both of the cells, potentially regulating the oxidative stress from the intracellular ROS species.

The DCFH-DA assay mainly measures H_2_O_2_ levels released by the cells. Co_3_O_4_ NPs showed higher fluorescence intensities because of their weaker ability in ROS inhibition. The CND treatment causing reduction in the fluorescence intensities could be explained by the antioxidant properties of CNDs by scavenging ROS radicals, as described in our previous findings.^6^ However, the Co_3_O_4_@CND hybrid NP reduction in the fluorescence intensities was expected because of the shell CNDs covering Co_3_O_4_.

#### 3.2.3 Cell Viability of Co_3_O_4_@CND Hybrid NPs

An AB assay was used to assess and compare the percentage viability of Co_3_O_4_@CND hybrid NPs, CNDs, and Co_3_O_4_ NPs in EAhy926 and A549 cells. The AB assay measurements revealed that the EAhy926 cell viability was around 77.1% and the A549 cell viability was around 56.6% at 0.8 mg/mL treated with the Co_3_O_4_@CND hybrid (Figure 6a,b). The results suggest that increasing the Co_3_O_4_@CND hybrid NP concentration caused higher cytotoxicity in cancer cells than in normal human cells.^31^ The Co_3_O_4_@CND hybrid NPs were more biocompatible than the Co_3_O_4_ NPs but less than the CNDs in the EAhy926 cells due to the biocompatibility of CNDs.^60^ The toxicity of the Co_3_O_4_@CND hybrid NPs at higher concentrations than CNDs is expected, given the toxic Co_3_O_4_ NPs inside them.^10,60^ However, to some extent, the hybrid NPs were still toxic to normal human cells at the same concentrations, requiring further modification.^10^

**Figure 6:**
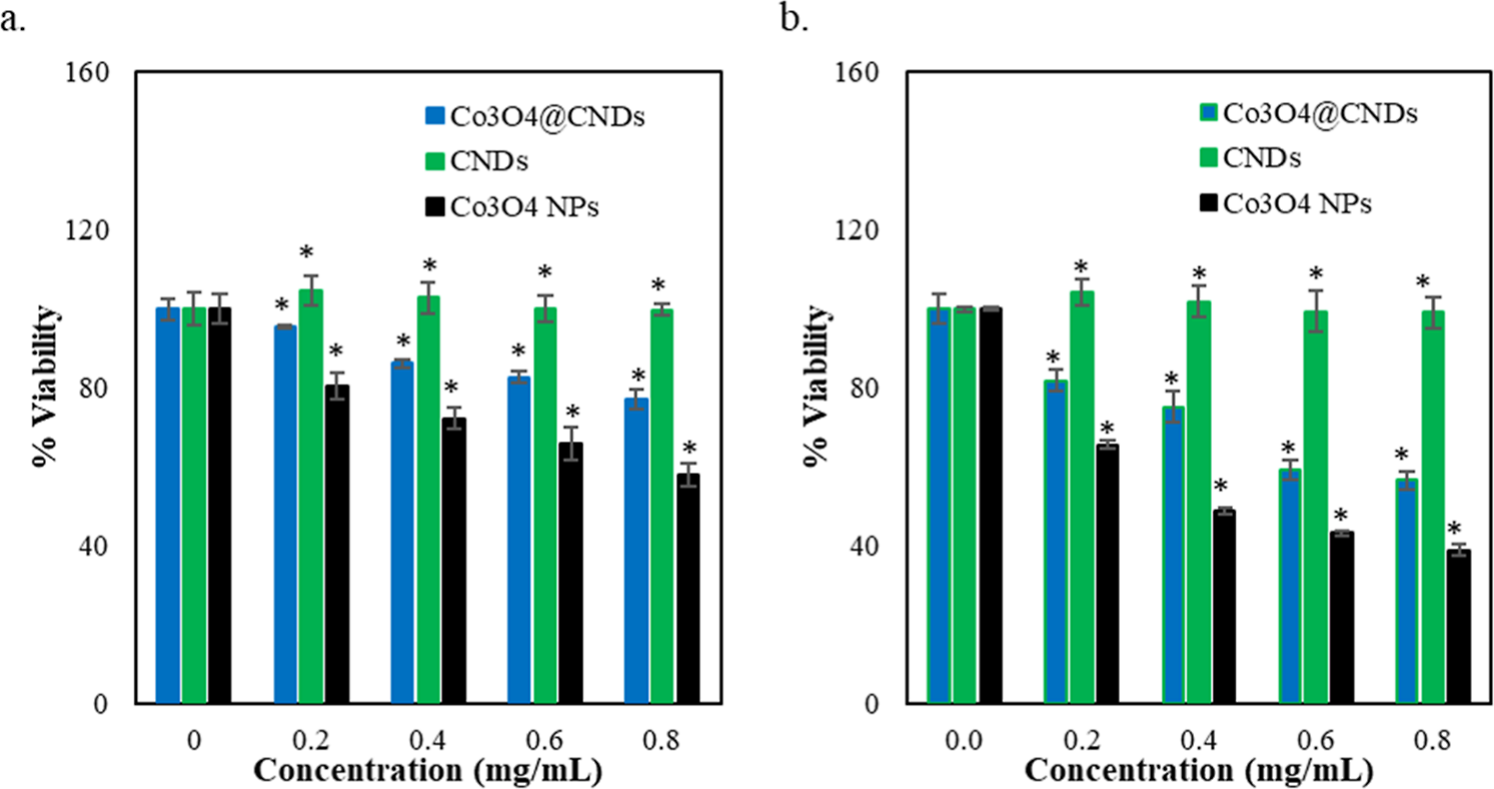
Cellular viability of the treatment with Co_3_O_4_@CND hybrid NPs, CNDs, and Co_3_O_4_ NPs in (a) EAhy926 and (b) A549 cells. Values were obtained as the mean ± SE from three independent experiments. Triplicates of each treatment were performed individually. *p < 0.05 versus control (0 mg/mL).

Additional tests, such as cellular uptake and ROS measurements, were performed to better comprehend the viability data and correlate with our data analysis.^40^ Based on these biological results, the Co_3_O_4_@CND hybrid NPs were designed to be biocompatible and cancer-specific simultaneously, as well as to improve the targeting of A549 cells by delivering them using ligands via ligands such as FA-BSA, Hep, Trf, and PEGylated SiO_2_, with or without DOX, which were attached to NPs using different synthesis approaches, as previously mentioned.^25,30,36,54^ To study and predict the cancer–targeting mechanisms of these NPs, determining how hybrid NPs reach the cancer cell receptors via different ligands might aid in the understanding and evaluation of potential anticancer mechanisms.

#### 3.2.4 Selective Cancer Cell Targeting of Ligand- and DOX-Conjugated Co_3_O_4_@CND Hybrid NPs Using Viability Studies

After characterization and understanding of the properties of the conjugated NPs, the percentage viabilities of FA-BSA-Co_3_O_4_@CND-DOX, Co_3_O_4_@CND-Hep-DOX, Co_3_O_4_@CND-Rhod, and Co_3_O_4_@CND-Trf-DOX were evaluated using AB assays in the EAhy926 (Figure S12a–d) and A549 (Figure S13a–d) cell lines. The results were compared with those of FA, DOX, FA-BSA-Co_3_O_4_@CNDs, Co_3_O_4_@CND-Hep, Hep, Co_3_O_4_@CND-Trf, Trf, Co_3_O_4_@CNDs, and DOX (Table S2).

The AB cell viability measurements showed that increasing the FA-BSA-Co_3_O_4_@CND-DOX NP concentration caused greater cytotoxicity in cancer cells than in EAhy926 cells (Figure 7). Moreover, FA-BSA-Co_3_O_4_@CND-DOX was not sufficiently toxic to A549 cells, similar to EAhy926 cells, which might be partly disadvantageous. Probably because FA has excellent biocompatibility but is not anticancerous itself, it serves as a better carrier for anticancer drugs.^61^ Instead of being cancer-specific only, free DOX showed extensive toxicity in the cell lines, particularly in the EAhy926 type.^37,54^ The FA-BSA ligand combined with Co_3_O_4_@CND NPs was toxic to normal human cells at higher concentrations. Therefore, the FA-BSA might not be an effective ligand for Co_3_O_4_@CNDs to target A549 cells.^37^ Other ligands were also tested using optimization to determine which receptors were overexpressed in A549 cancer cells specifically.^1^ Hep-NHS was safe for EAhy926 and A549 cells in its native form and when combined with Co_3_O_4_@CNDs (Figures S12b and S13b). The viability results showed that at increasing concentrations of Hep-Co_3_O_4_@CND-DOX NPs exhibited more cytotoxicity in EAhy926 cells than in A549 cells. Moreover, Hep-Co_3_O_4_@CNDs were not toxic enough to A549 cancer cells, even a little more toxic than EAhy926 cells. It is reported Hep ligands target the overexpressed CD44 and heparin-binding growth factor receptors.^7,62^ Our results indicate anticancer properties were improved by conjugating DOX.^7^ It was observed that the anticancer effect of Co_3_O_4_@CNDs-Hep-DOX was greater than that of Co_3_O_4_@CNDs because of DOX’s prohibition of cancer cell proliferation.^7^ However, the Co_3_O_4_@CNDs-Hep-DOX conjugates considerably affected human cells too. At higher concentrations, the combination of the Hep ligands and DOX with Co_3_O_4_@CND NPs was more cytotoxic to human cells than to A549 cells. Owing to this adverse effect, Hep might not be an effective ligand for Co_3_O_4_@CNDs to target A549 cells.

**Figure 7:**
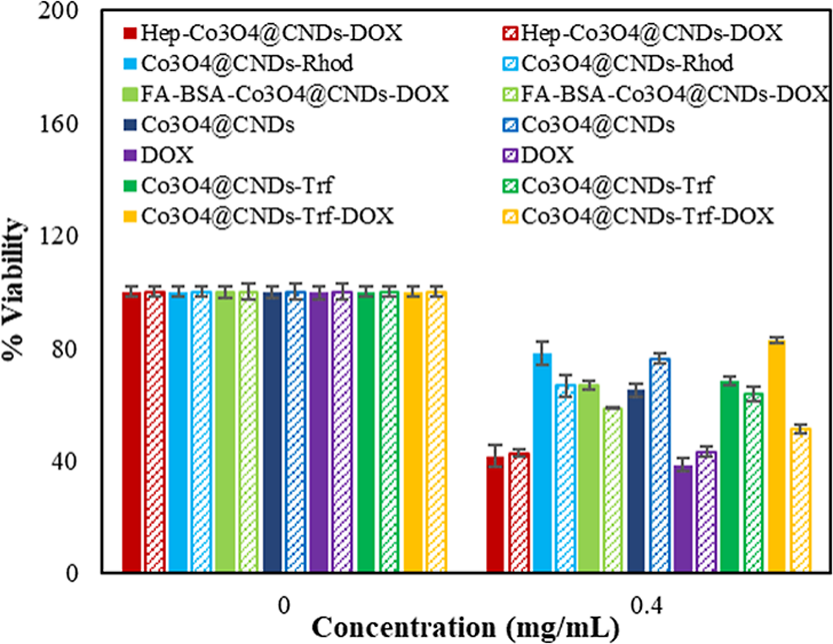
Percentage viability in EAhy926 (solid-filled) and A549 cells (pattern-filled) for Co_3_O_4_@CNDs with different conjugations.

The viability results (Figures S12c and S13c) showed that increasing concentrations of Co_3_O_4_@CND-Rhod exhibited greater cytotoxicity in A549 cells than in EAhy926 cells. At a concentration of 0.4 mg/mL, the viabilities of Co_3_O_4_@CNDs-Rhod in EAhy926 and A549 cells were 65.02% and 76.31%, respectively. Thus, Co_3_O_4_@CNDs-Rhod was more biocompatible and anticancerous to A549 cells than Co_3_O_4_@CNDs. For this reason, PEG-SiO_2_ (Rhod) might be considered an effective ligand for Co_3_O_4_@CNDs to target A549 cells, while they had a lower anticancerous effect than FA-BSA-Co_3_O_4_@CNDs-DOX. The anticancer mechanism of these conjugates was thought to be energy-dependent endocytosis and phagocytosis, in which the ligands are transported, internalized, and absorbed by cancer cells.^22^ In addition, the Co_3_O_4_@CNDs-Rhod particles might target folate receptors that are overexpressed on the surface of cancer cells because of the PEG and SiO_2_ functionalization of the Co_3_O_4_@CNDs.^63^

Figures S12d and S13d show that increasing concentrations of Co_3_O_4_@CND-Trf-DOX exhibited greater cytotoxicity in A549 cells than in EAhy926 cells. The Trf ligand and DOX conjugation with Co_3_O_4_@CND NPs were more cytotoxic to the cancer cells at higher concentrations; Trf was safe for normal human cells, while DOX was more toxic. Therefore, Trf was an effective ligand for the Co_3_O_4_@CNDs to specifically target A549 cells. The anticancer property of Trf-loaded Co_3_O_4_@CND NPs was increased via modification with DOX. Trf-Co_3_O_4_@CNDs-DOX caused a decrease in cytotoxicity (51.16%) compared to Trf (59.63%), Trf-Co_3_O_4_@CNDs (64.03%), and Co_3_O_4_@CNDs (65.02%) after 24 h of incubation with A549 cells at a concentration of 0.4 mg/mL. The literature suggests that the anticancer mechanism of this ligand is to target specific overexpressed Trf and Trf1 receptors on the surface of A549 cancer cells, resulting in endocytosis via the designed complex.^26,53,54^

### 3.3 Discussion

The cell viability, anticancer properties, bioimaging, and antioxidant behavior of the Co_3_O_4_@CND hybrid NPs and those with different bioconjugations were assessed and compared with their counterparts. The bioimaging results showed that the Co_3_O_4_@CND hybrid NPs were superior to CNDs only. Cancer cell specificity was based on different ligands using viability assays, and the effects of each ligand were compared by targeting A549 LC cells. The viability comparison results showed that the Rhod– and Trf-DOX–conjugated Co_3_O_4_@CND hybrid NPs were more selective targeting ligands for A549 cancer cells and less toxic to EAhy926 normal cells.

Table S2 summarizes a comparison for a better understanding of the anticancer effects of all conjugated ligands with Co_3_O_4_@CND hybrid NPs. Trf-DOX, Co_3_O_4_@CNDs-Rhod, and FA-BSA-DOX conjugation with Co_3_O_4_@CND hybrid NPs were more effective for targeting conjugates for A549 cells than Hep-DOX, reducing the toxicity to Eahy926 cells and improving their specificity and targeting.

Figure 8 shows the proposed anticancer mechanism of the Co_3_O_4_@CND hybrid NPs with ligands and drugs loaded on them. The ligands FA-BSA, Hep, PVP-PEG-SiO_2_-Rhod, and Trf as well as the anticancer drug DOX target receptors in A549 lung cancer cells including folate, FGFR, Trf1, alpha 5, beta 3 (α_v_β_3_) integrin, hepatocyte GFR (HGFR), G-protein coupled, CXCR4, EGFR, and CD44.^21,24,64–66^ The anticancer activity was expected because of the active targeting of ligands via receptor activation.^1^ The active targeting of the A549 cancer cells is achieved by complete receptor–ligand complex activation for NP delivery combined with the anticancer drug DOX. Once the receptors on the A549 cancer cell surface are activated, the NPs and drugs are released inside the cells via endocytosis or pinocytosis, inducing lysosomal degradation, which further leads to apoptosis and cell death.^6,64,66^ The Co_3_O_4_@CND hybrid NPs with Trf and DOX are more specific and targeted without affecting normal (Eahy926) cells, indicating that Trf1 and CD44 receptors are more specific targeting in A549 cancer cells,^54^ and then release the NPs, increasing the anticancer activity. In addition, the ligand Trf was more biocompatible than the other ligands used, which is beneficial for delivering the NP.^54^

**Figure 8:**
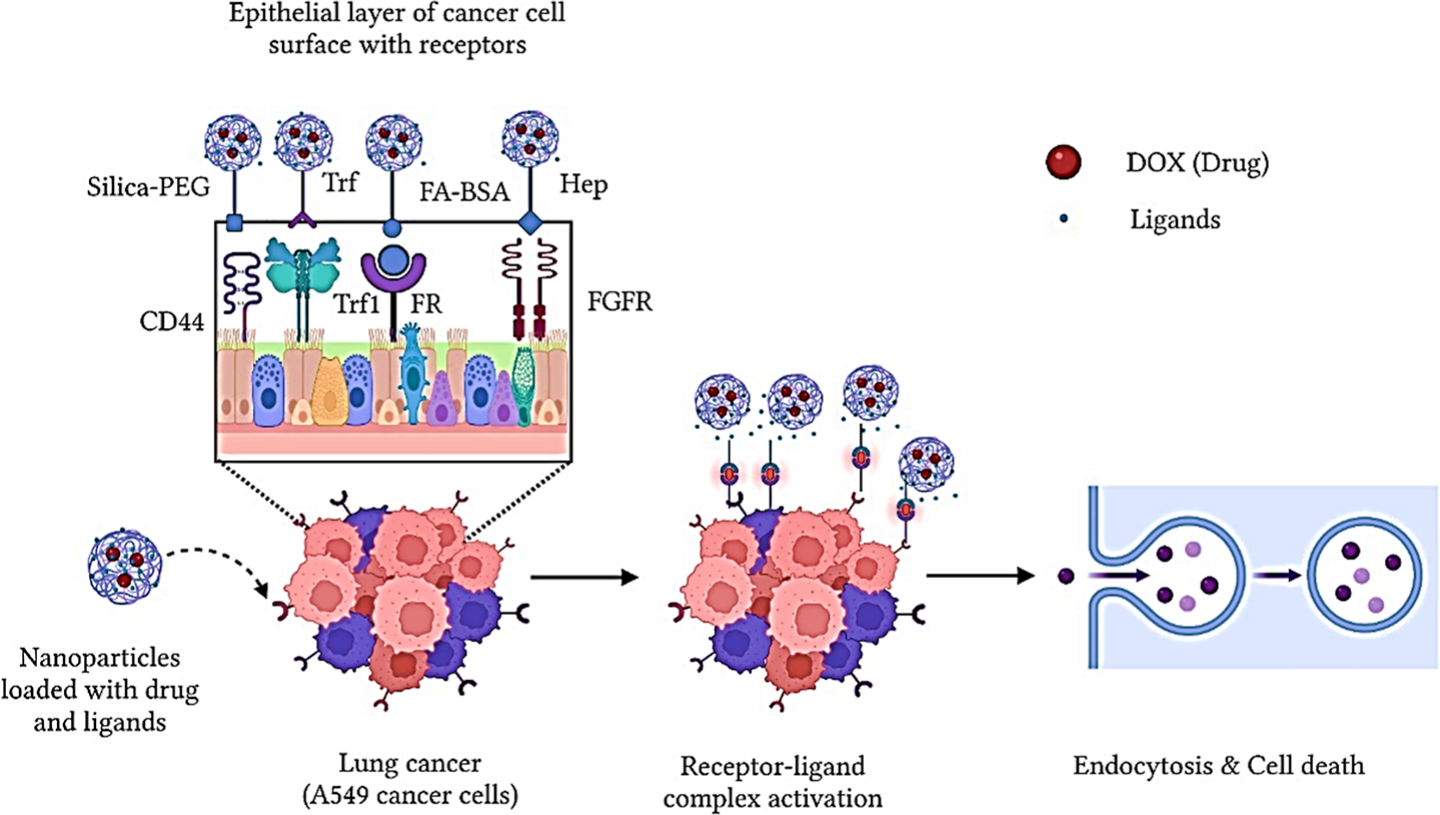
Illustration of the proposed anticancer mechanism for ligand– and DOX–conjugated Co_3_O_4_@CND hybrid NPs.

Table S3 summarizes a comparison of DOX-based inorganic nanocomposites used and researched for theranostic applications in A549 cells with our current work. To this end, one can conclude that the Co_3_O_4_@CND hybrid NPs potentially serve as multifunctional theranostic candidates for further studies. The previous study found that the CND uptake in human and cancer cells mostly occurred by macropinocytosis and lipid-raft–mediated endocytosis.^6^ We believe that the Co_3_O_4_@CND hybrid NPs might follow similar endocytosis mechanisms due to the similar CND structure on the outside of the hybrid NPs. However, additional investigations are needed.

## 4 Conclusions

Co_3_O_4_@CND hybrid NPs were studied in this research for their advanced multifunctional applications, including anticancer, bioimaging, antioxidant, and drug delivery carriers. This study suggests that the Co_3_O_4_@CND hybrid NPs possess anticancer and antioxidant activity, as well as an efficient imaging probe with brighter fluorescence than the CNDs. Furthermore, advanced active targeting strategies were demonstrated by loading anticancer drug (DOX) for enhanced anticancer activity and improving the specificity to target A549 cancer cells via ligand conjugation. The anticancer activity was tested for each ligand-conjugated NP and compared in the EAhy926 and A549 cancer cells. By comparing four distinct ligand-conjugated NPs, we inferred that the Trf-DOX conjugate with Co_3_O_4_@CND hybrid NPs possessed superior anticancer activity with enhanced biocompatibility. The Co_3_O_4_@CND hybrid NPs with ligand conjugation are potential multifunctional theranostic agents with high biocompatibility and high drug-loading capabilities.

## Supplementary Material



## Notes

Quantum yield, FTIR, excitation dependency, and XPS data; characterization of the conjugated ligands with Co_3_O_4_@CND hybrid NPs; confocal microscopic analysis data; antioxidant analysis; ζ potential table for conjugates; and cell viability results of the conjugated ligands with Co_3_O_4_@CND hybrid NPs and their respective comparison table (PDF)
