# Supplementary material for: Multifunctional Core–Shell Cobalt Oxide @ Carbon Nanodot Hybrid Conjugates for Imaging and Targeting A549 Cells
Source: ACS Appl Bio Mater. 2025 Jun 5;8(6):4981–94. doi: 10.1021/acsabm.5c00343 (PMC12175123; doi:10.1021/acsabm.5c00343)
Supplement: Supplementary file 1 [file mt5c00343_si_001.pdf]

Supporting Information

Multifunctional Core-Shell Cobalt Oxide @ Carbon Nanodot Hybrid Conjugates for Imaging and Targeting A549 Cells

*Anitha Jayapalan, Frank Tukur, Mahsa Azami, Mengxin Liu, and Jianjun Wei**

The Department of Nanoscience, Joint School of Nanoscience and Nanoengineering, The University of North Carolina at Greensboro,

2907 E. Gate City Blvd, Greensboro, NC 27401, USA

*Corresponding Author: j_wei@uncg.edu

Table of Content

Figure S1. Synthesis schematic and a photograph of synthesized NPs in vials under UV light. S3

Figure S2. FTIR of a. FA-BSA-Co_3O_4@CND-DOX, b. Hep-Co_3O_4@CND-DOX, c. Co_3O_4@CND-Rhod, and d. Co_3O_4@CND-Trf-DOX hybrids. S4

Figure S3. UV-Visible Spectra of a. FA-BSA-Co_3O_4@CND-DOX, b. Hep-Co_3O_4@CND-DOX, c. Co_3O_4@CND-Rhod, and d. Co_3O_4@CND-Trf-DOX hybrids. S5

Figure S4. PL Spectra of a. FA-BSA-Co_3O_4@CND-DOX, b. Hep-Co_3O_4@CND-DOX, c. Co_3O_4@CND-Rhod, and d. Co_3O_4@CND-Trf-DOX hybrids. S6

Figure S5. Excitation-dependency emission spectra of a. CNDs, b. Co_3O_4@CNDs, c. FA-BSA-Co_3O_4@CND-DOX, d. Hep-Co_3O_4@CND-DOX, and e. Co_3O_4@CND-Trf-DOX hybrids. S7

Figure S6. Zeta Potential data of a. FA-BSA-Co_3O_4@CND-DOX, b. Hep-Co_3O_4@CND-DOX, c. Co_3O_4@CND-Rhod, and d. Co_3O_4@CND-Trf-DOX hybrids. S8

Figure S7. Cellular uptake of Co_3O_4@CNDs with increased concentrations in EAhy926 cells at 63X magnification. S9

Figure S8. Cellular uptake of Co_3O_4@CNDs with an increase in concentrations in A549 cells at 63X magnification. S10

Figure S9. Confocal images of **s**ub-cellular localization of CNDs in EAhy926 and A549 cells at 100X magnification. S11

Figure S10. Fluorescence intensity plot profiles of Co_3O_4@CND hybrid NPs in a. EAhy926 and b.A549 cells. S12

Figure S11. DCFH-DA comparison plots of Co_3O_4@CND hybrid NPs in EAhy926 and A549 cells. S13

Figure S12. Percentage viability of EAhy926 cells with a. FA-BSA-Co_3O_4@CND-DOX, b. Hep-Co_3O_4@CND-DOX, c. Co_3O_4@CND-Rhod, and d. Co_3O_4@CND-Trf-DOX. S14

Figure S13. Percentage viability plots of A549 cells with a. FA-BSA-Co3O4@CND-DOX, b. Hep-Co3O4@CND-DOX, c. Co3O4@CND-Rhod, and d. Co3O4@CND-Trf-DOX hybrids. S15

Table S1. The zeta potential of all the conjugated particles. S16

Table S2. Comparison analysis of percentage viability in EAhy926 and A549 cells for Co_3O_4@CND's conjugates. S17

Table S3: Comparison of DOX-based inorganic nanocomposites used and researched for theranostic applications in A549 cells. S18

Reference. S20

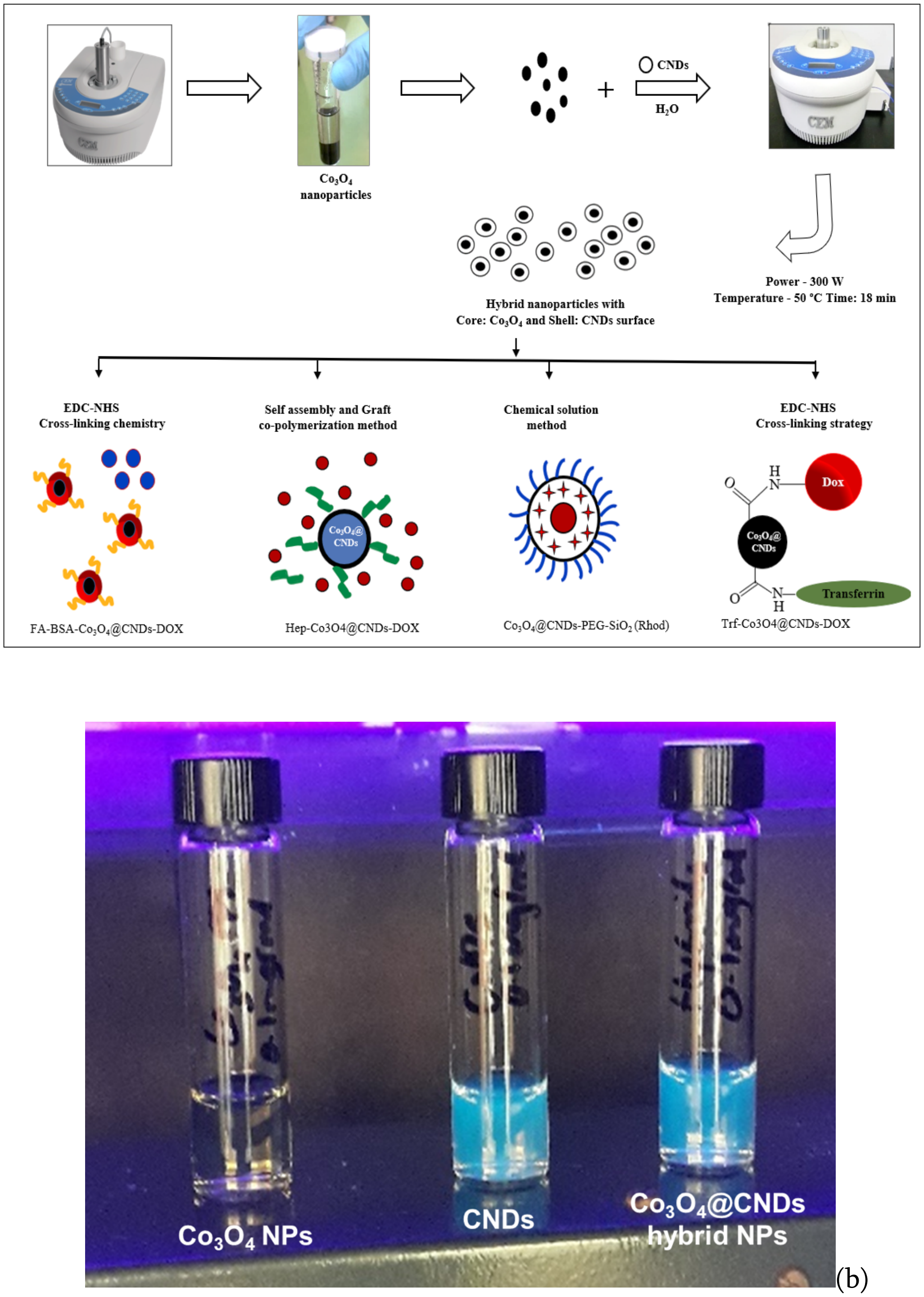

(a)

(b)

Figure S1. a). Schematic of the synthesis process, b). A photograph of synthesized NPs in vials under UV light.

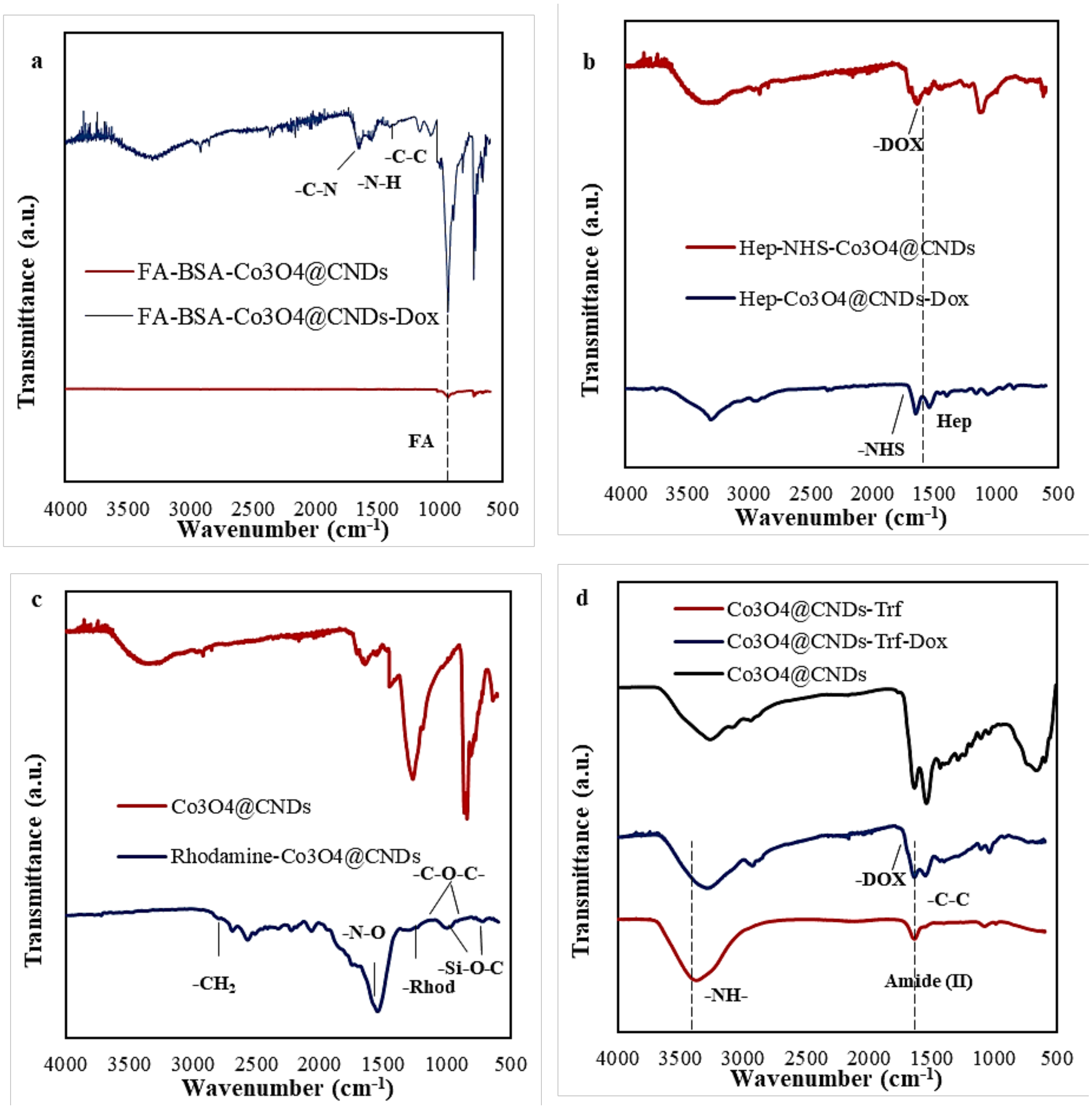

Figure S2. FTIR of a). FA-BSA-Co_3O_4@CND-DOX, b). Hep-Co_3O_4@CND-DOX, c). Co_3O_4@CND-Rhod, and d). Co_3O_4@CND-Trf-DOX hybrids.

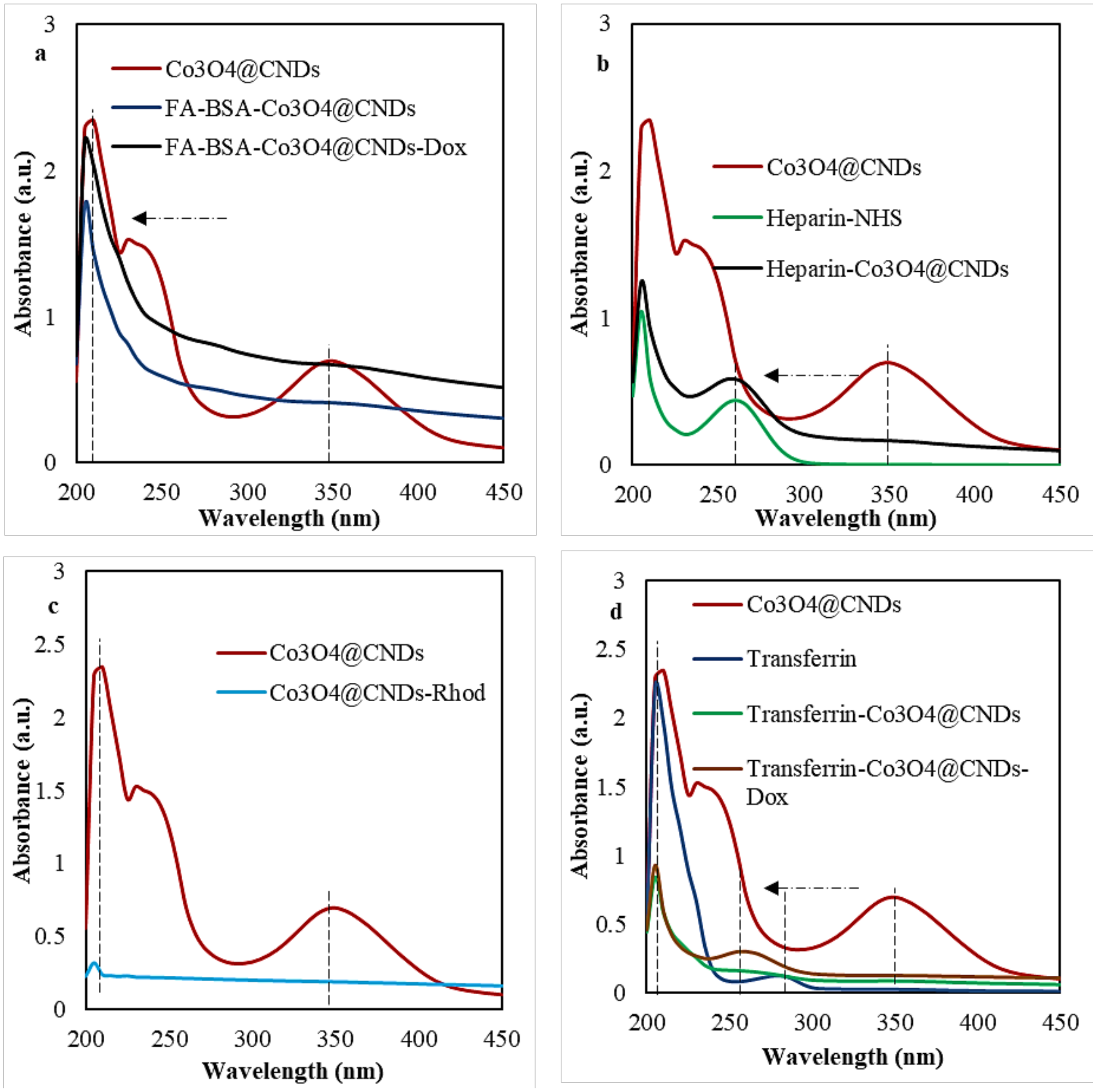

Figure S3. UV-Visible Spectra of a). FA-BSA-Co_3O_4@CND-DOX, b). Hep-Co_3O_4@CND-DOX, c). Co_3O_4@CND-Rhod, and d). Co_3O_4@CND-Trf-DOX hybrids.

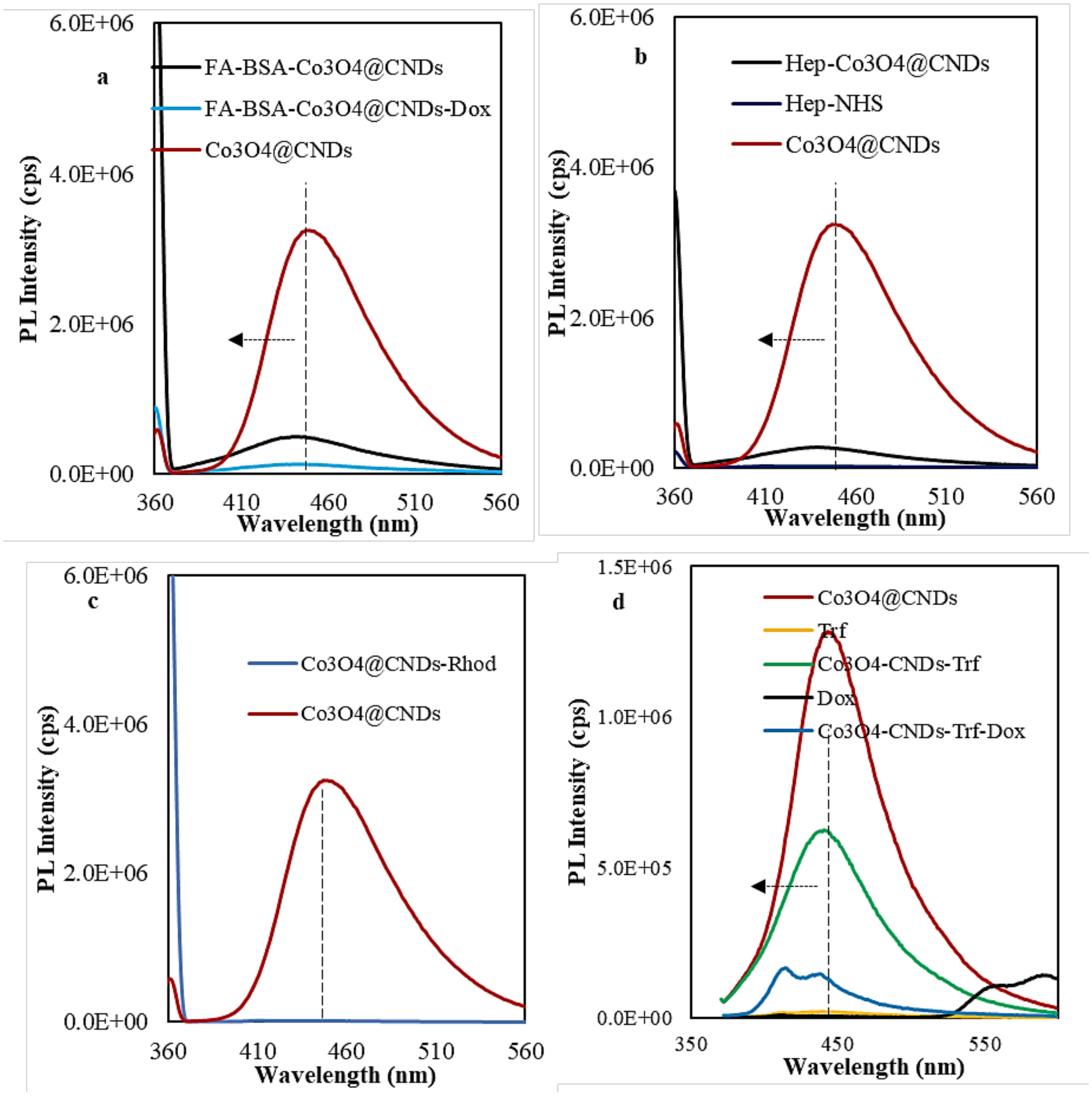

Figure S4. PL Spectra of a). FA-BSA-Co_3O_4@CND-DOX, b). Hep-Co_3O_4@CND-DOX, c). Co_3O_4@CND-Rhod, and d). Co_3O_4@CND-Trf-DOX hybrids.

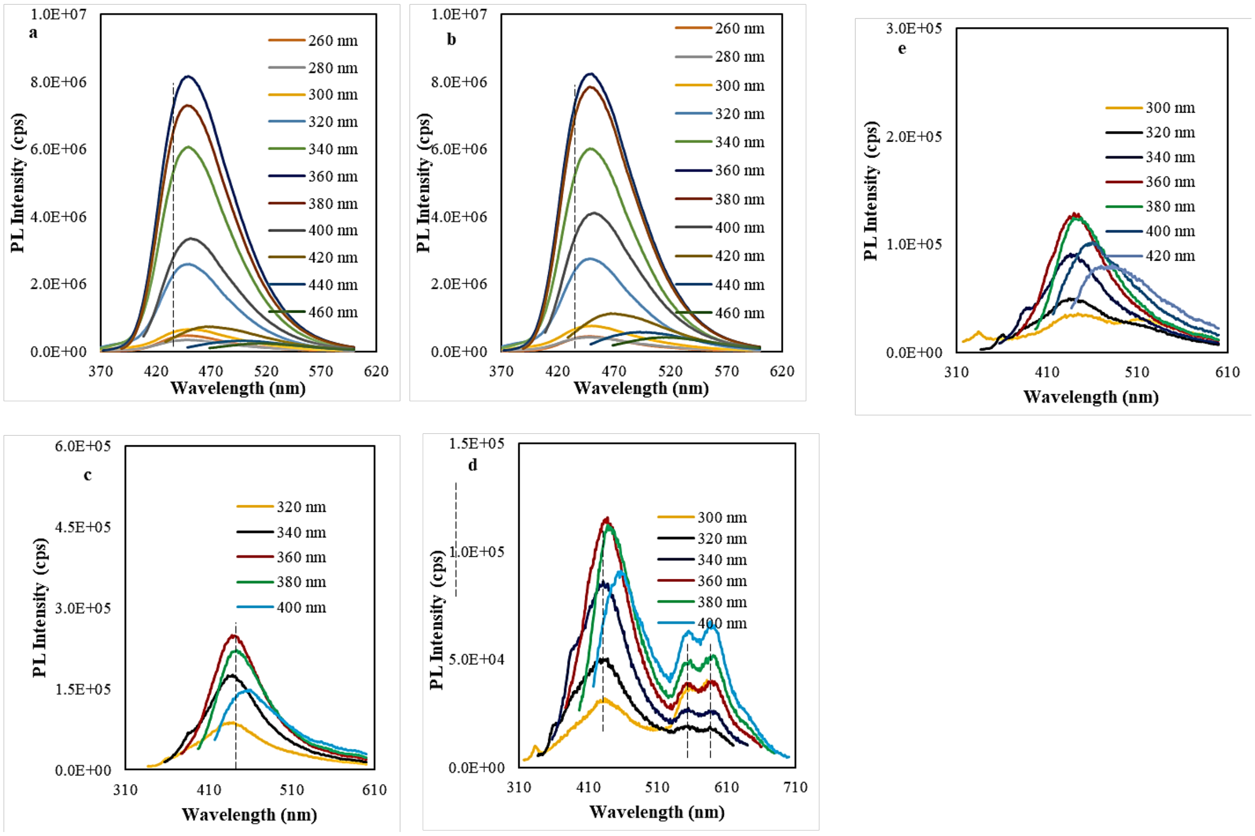

Figure S5. Excitation-dependency emission spectra of a). CNDs, b). Co_3O_4@CNDs, c). FA-BSA-Co_3O_4@CND-DOX, d). Hep-Co_3O_4@CND-DOX, and e). Co_3O_4@CND-Trf-DOX hybrids.

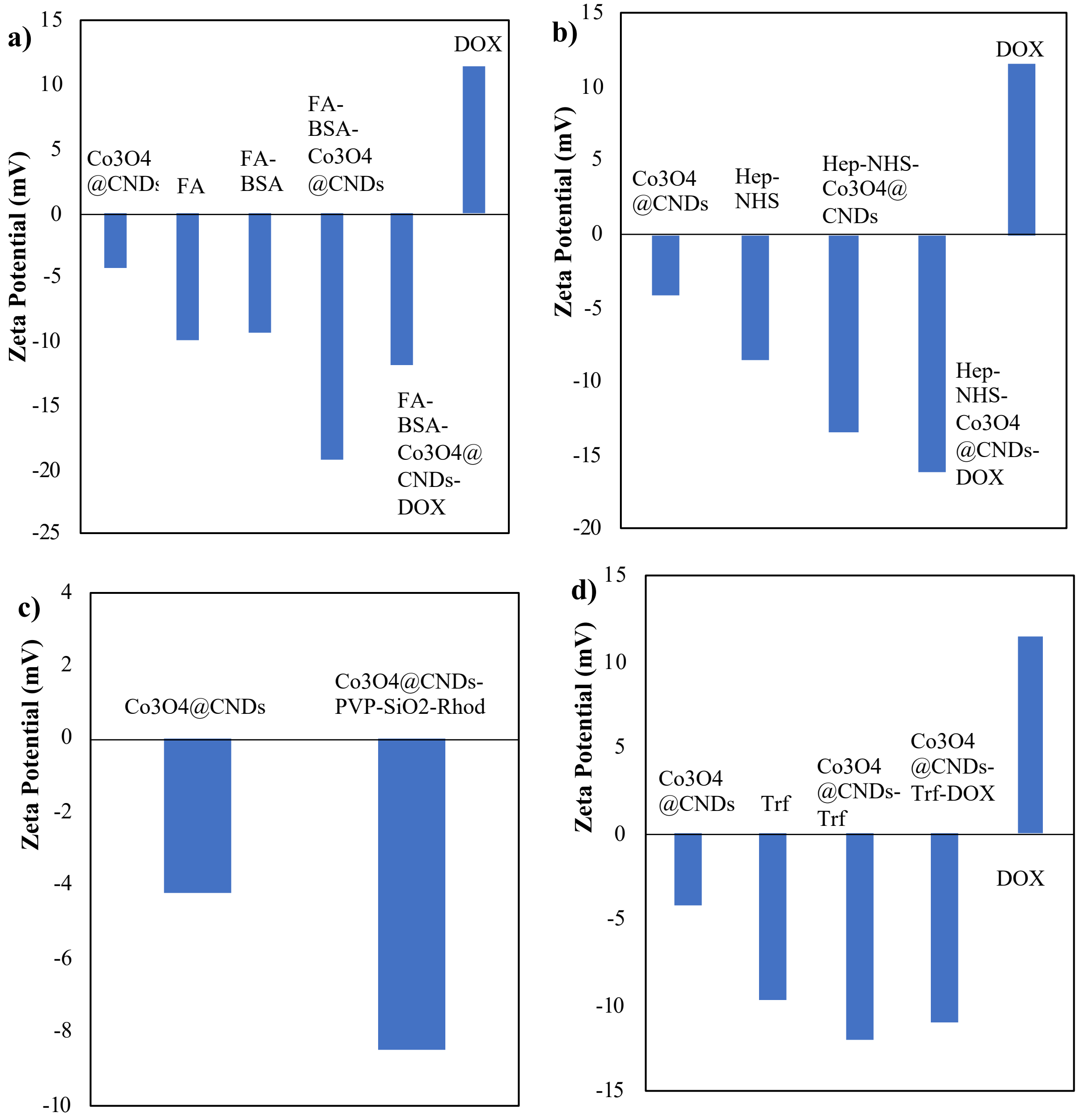

Figure S6. Zeta Potential data of a). FA-BSA-Co_3O_4@CND-DOX, b). Hep-Co_3O_4@CND-DOX, c). Co_3O_4@CND-Rhod, and d). Co_3O_4@CND-Trf-DOX hybrids.

Confocal microscopy results in comparison at different concentrations at 63X magnification

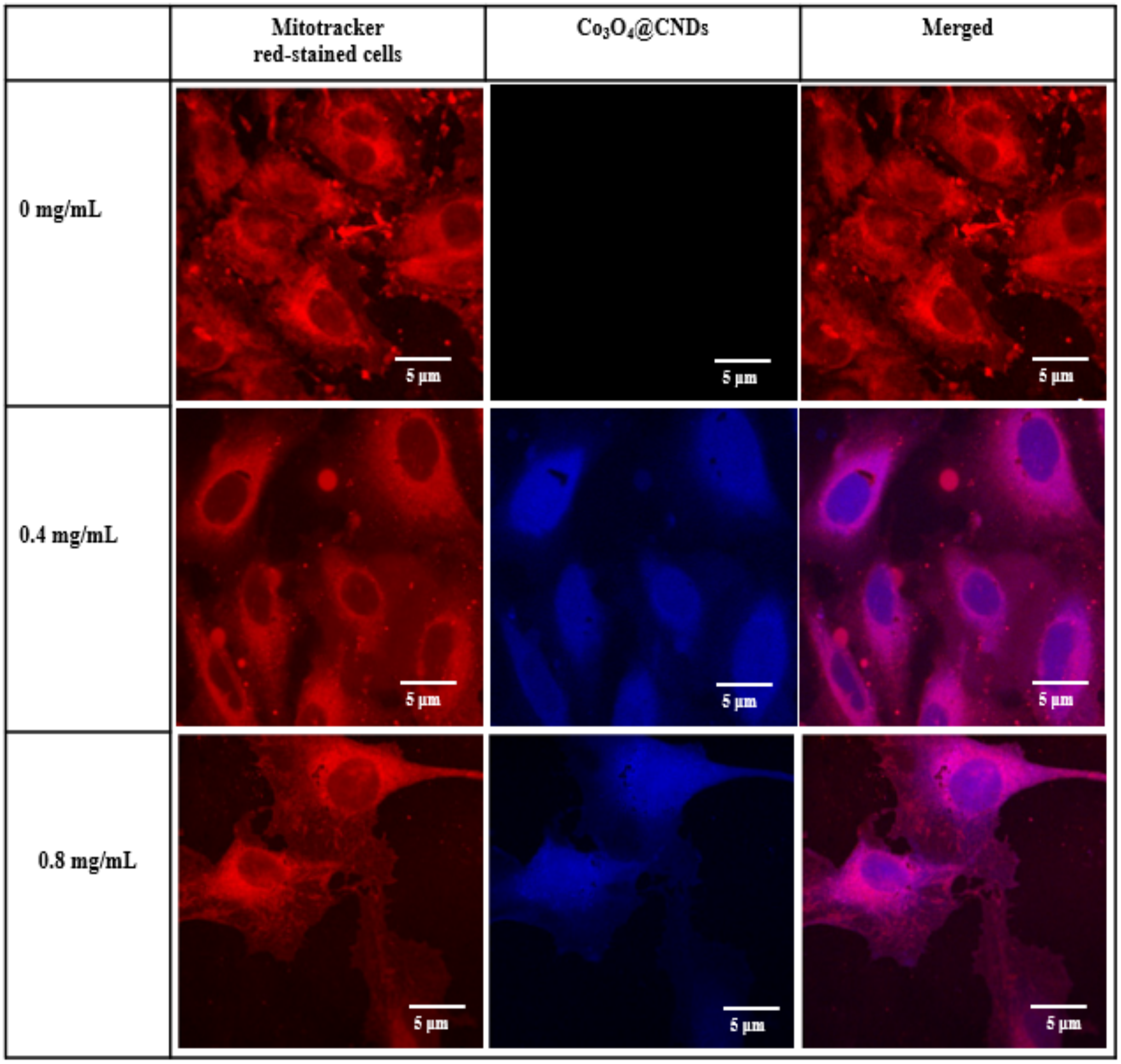

Figure S7. Cellular uptake of Co_3O_4@CNDs with increased concentrations in EAhy926 cells at 63X magnification.

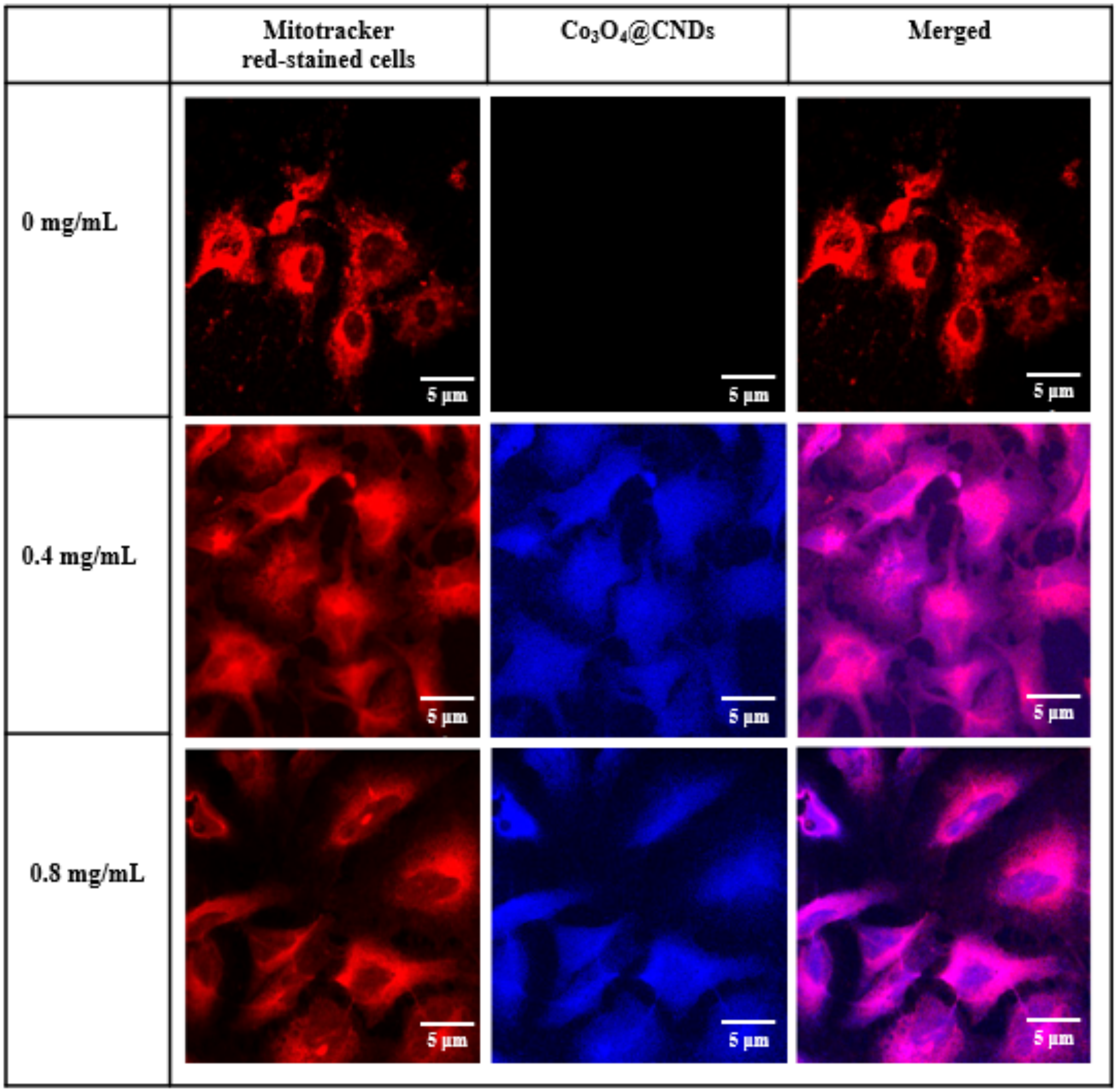

Figure S8. Cellular uptake of Co_3O_4@CNDs with an increase in concentrations in A549 cells at 63X magnification.

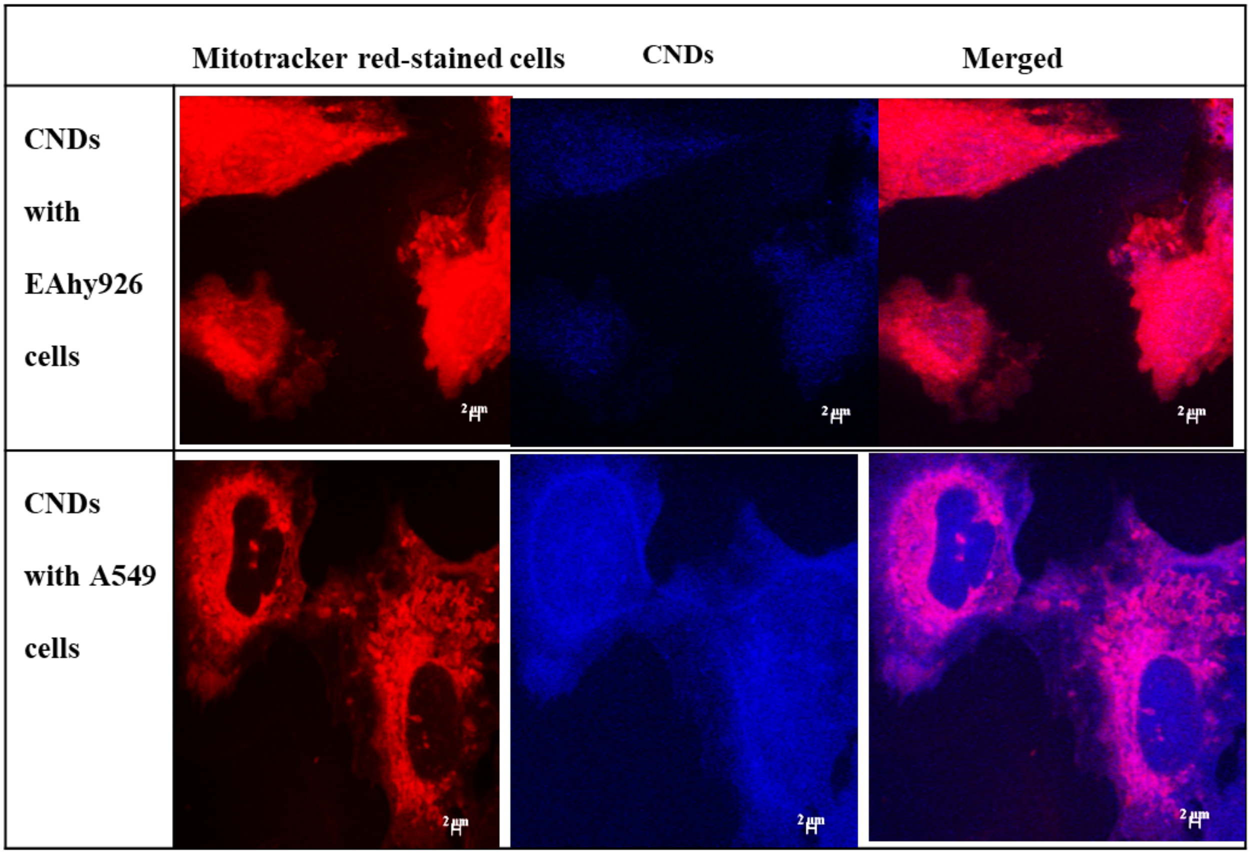

Figure S9. Confocal images of sub-cellular localization of CNDs in EAhy926 and A549 cells at 100X magnification.

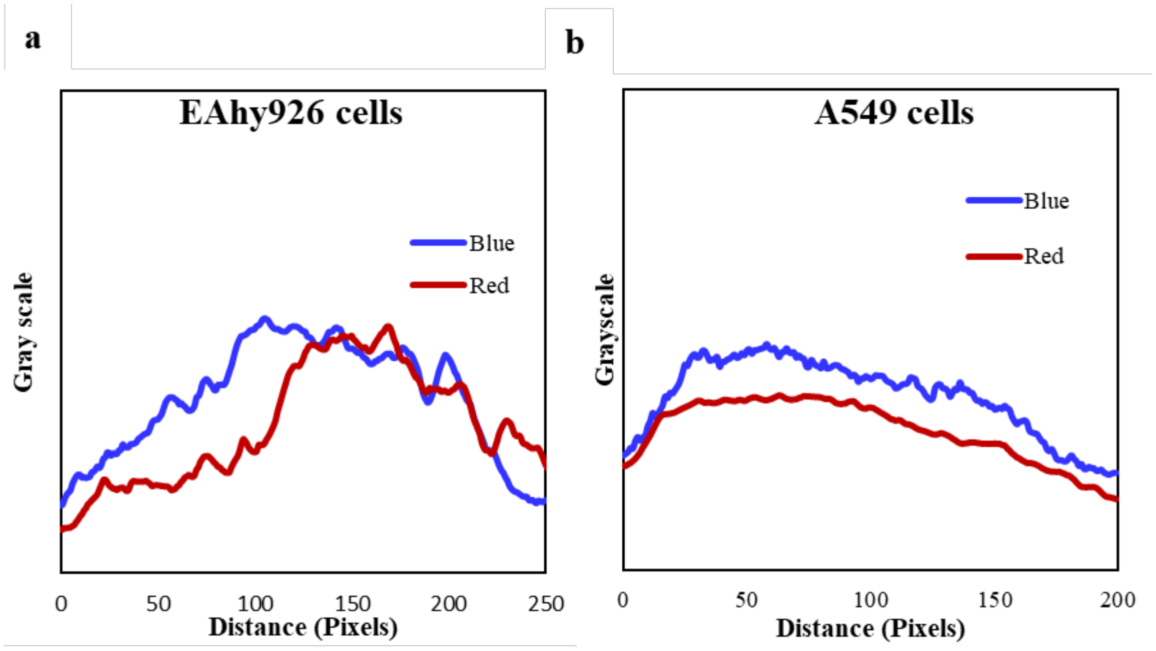

Figure S10. Fluorescence intensity plot profiles of Co_3O_4@CND hybrid NPs in a. EAhy926 and b.A549 cells.

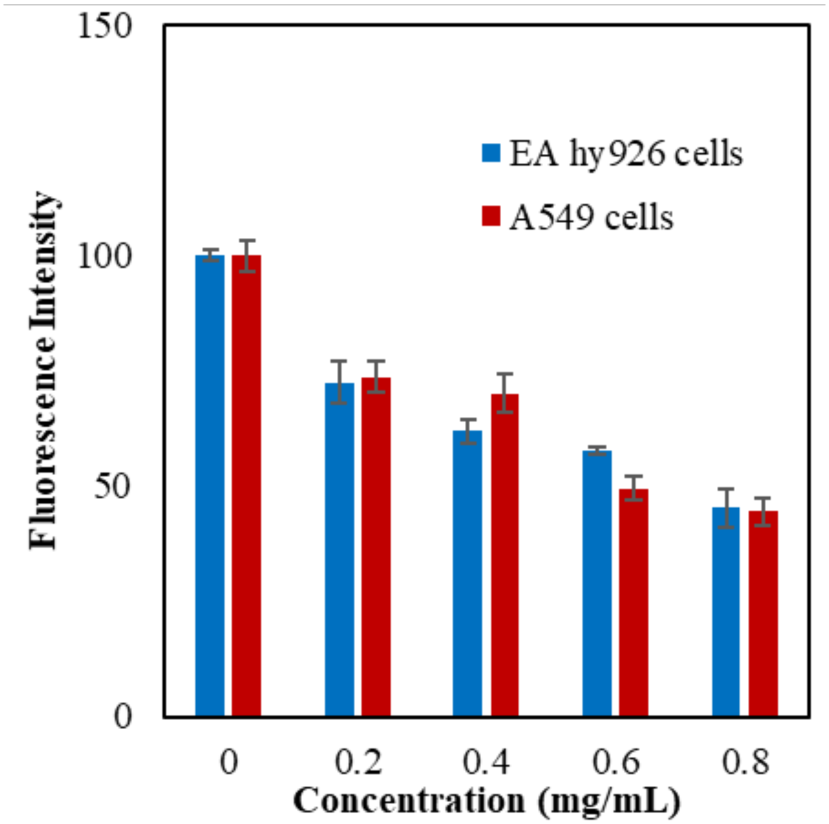

Figure S11. DCFH-DA comparison plots of Co_3O_4@CND hybrid NPs in EAhy926 and A549 cells.

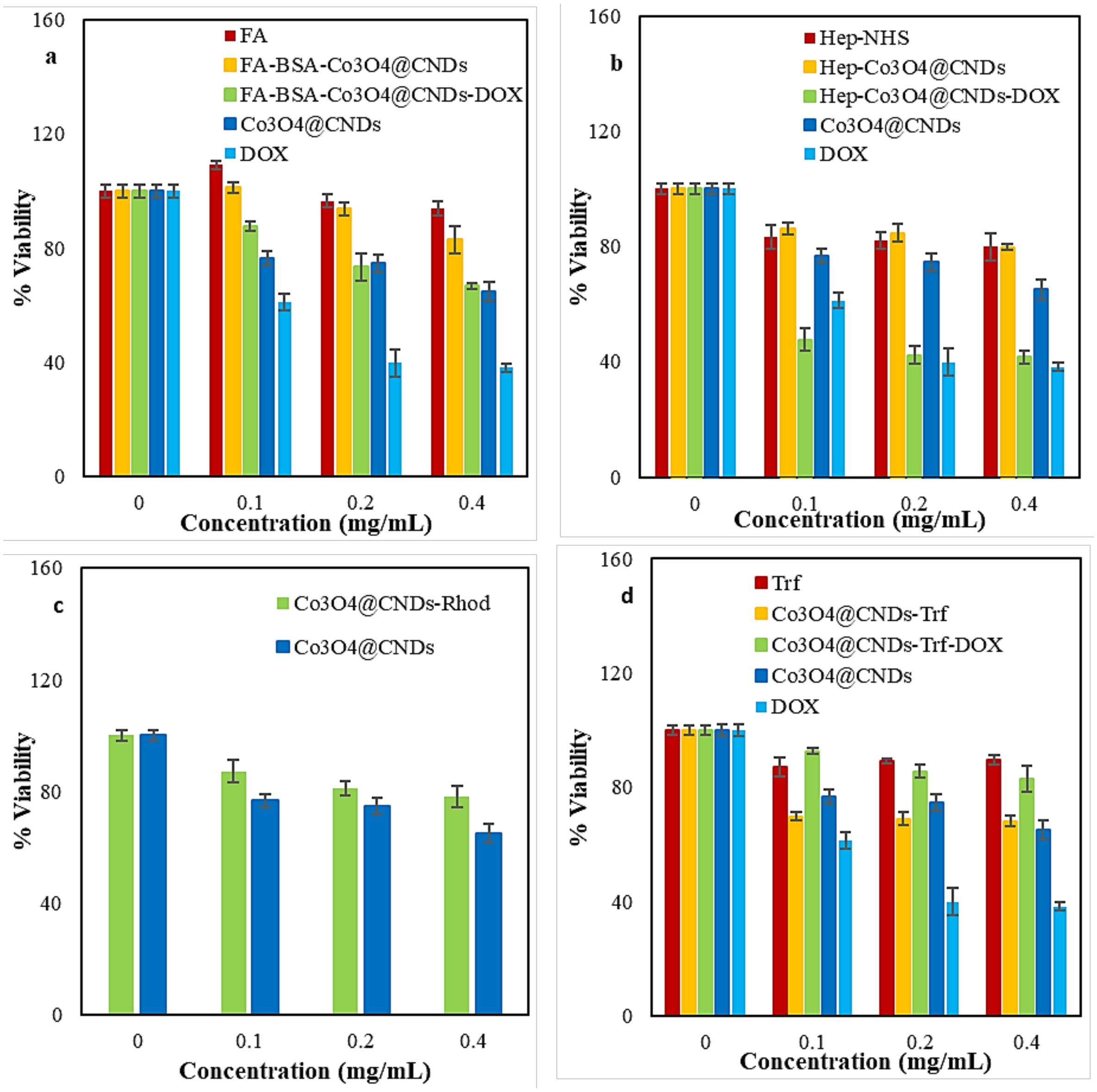

Figure S12. Percentage viability of EAhy926 cells with a. FA-BSA-Co_3O_4@CND-DOX, b. Hep-Co_3O_4@CND-DOX, c. Co_3O_4@CND-Rhod, and d. Co_3O_4@CND-Trf-DOX.

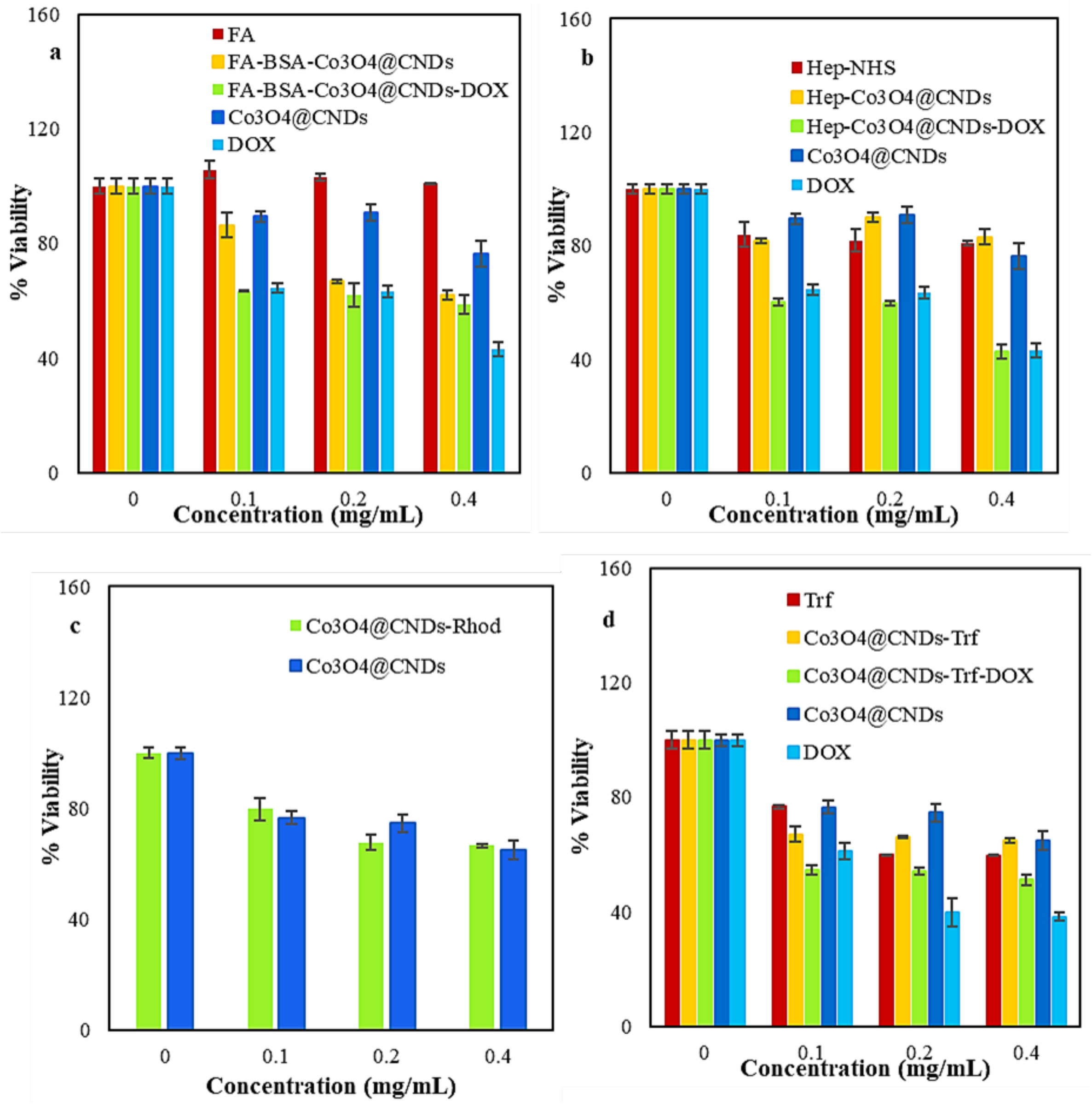

Figure S13. Percentage viability plots of A549 cells with a. FA-BSA-Co3O4@CND-DOX, b. Hep-Co3O4@CND-DOX, c. Co3O4@CND-Rhod, and d. Co3O4@CND-Trf-DOX hybrids.

Zeta potential table for the conjugated particles along with the ligands

Table S1 The zeta potential of all the conjugated particles.

**Samples**	**Zeta Potential**
Co_3O_4@CNDs	-4.19±0.11 Mv
FA	-9.81±0.43 mV
FA-BSA	-9.30±0.36 mV
FA-BSA-Co_3O_4@CNDs	-19.13±1.23 mV
FA-BSA-Co_3O_4@CNDs-DOX	-11.8±0.8 mV
Hep-NHS	-8.52±0.24 mV
Hep-NHS-Co_3O_4@CNDs	-13.47±0.27 mV
Hep-NHS-Co_3O_4@CNDs-DOX	-16.1±0.9 mV
Co_3O_4@CNDs-Rhod	-8.52±0.24 mV
Trf	-9.63±0.29 mV
Co_3O_4@CND-Trf	-11.97±0.53 mV
Co_3O_4@CND-Trf-DOX	-11.03±0.73 mV
DOX	11.53±0.77 mV

Conjugation cell viability - pros and cons.

Table S2. Comparison analysis of percentage viability in EAhy926 and A549 cells for Co_3O_4@CND's conjugates.

**Conjugated targeting ligands with Co_3O_4@CND hybrid nanoparticles**	**Pros**	**Cons**
FA-BSA-Doxorubicin	More cancer specific<br>Viability at 0.4 mg/mL: 58.76%	At 0.4 mg/mL concentration, viability: 66.83%
Heparin-Doxorubicin	Viability for A549 cells at 0.4 mg/mL: 42.66%	Not much safe to EA hy926 cells; toxic at 0.4 mg/ml concentration, viability: 41.61% and at 0.1 mg/mL
Rhodamine conjugation	Viability for EAhy926 cells at 0.4 mg/mL: 78.15%<br>Viability for A549 cells at 0.4 mg/mL: 66.54%	
Transferrin-Doxorubicin	Viability for EAhy926 cells at 0.4 mg/mL: 82.86%<br>Viability for A549 cells at 0.4 mg/mL: 51.19%	
**Controls:**<br>Co_3O_4@CND hybrid nanoparticles only – no conjugation		Viability for EAhy926 cells at 0.4 mg/mL: 65.02%<br>Viability for A549 cells at 0.4 mg/mL: 76.31%
Doxorubicin only - no conjugation		Viability for EAhy926 cells at 0.4 mg/mL: 38.25%<br>Viability for A549 cells at 0.4 mg/mL: 43.16%

Table S3: Comparison of DOX-based inorganic nanocomposites used and researched for theranostic applications in A549 cells.

S.No.	Nanocomposite	Imaging Capability	Drug Loading Efficiency	Therapeutic Action	Key Challenges/ Remarks	Ref.
1.	Fe_3O_4@SiO_2@DOX-FA	MRI	Moderate (~55%)	Targeted chemotherapy	Agglomeration without PEGylation	1
2.	Au@BSA-DOX	CT imaging (weak) + some fluorescence	Moderate (~50–60%)	Chemotherapy + photothermal	Rapid clearance by macrophages; moderate toxicity to normal cells	2
3.	ZnO@BSA-Dox	UV fluorescence	Moderate (~50–65%)	pH-responsive drug release	UV excitation needed; moderate to high cytotoxicity to normal cells	3
4.	CuO@Dextran-Dox	Minimal (no imaging)	~55–60%	ROS-mediated cytotoxicity	High toxicity to healthy cells	4
5.	TiO_2@PEG-FA	UV-induced fluorescence	Moderate (~60%)	Photodynamic therapy + targeted drug delivery	Requires UV activation; limited tissue penetration	5
6.	AuNR-S-PEG.β-CD NS-DOX	NIR-responsive	Moderate (~60%)	Chemo-photothermal therapy	Limited penetration depth of NIR light	6
7.	HPDA/Au@DOX	Photothermal imaging	High (>80%)	Chemo-photothermal therapy	Potential aggregation of nanoparticles	7

8.	Graphene oxide nanosheets@DOX	Fluorescence (quenching effect)	High (~75%)	Drug delivery + photothermal therapy	Moderate toxicity to normal cells; Potential long-term toxicity; aggregation issues	8
9.	Fe_3O_4@SiO_2-Trf-DOX	MRI	High (~85%)	Dual-targeted drug delivery	Potential immunogenicity of silica covering and magnetic radiations to normal cells	9
10.	CNDs	Optical bioimaging	-	Antioxidant and anticancer effects	More sensitive to normal cells than cancer cells	10
11.	Co_3O_4@CND hybrid NPs-Trf-DOX	Optical bioimaging	High (~98%)	Better anticancer property; Less toxicity to normal cells, More bioimaging capability to EAhy926 and A549 cells. More uniform and smaller in size Enhanced therapeutic synergy and multifunctional platform use.	-	This study

References:

(1) Huang, Y.; Mao, K.; Zhang, B.; Zhao, Y. Superparamagnetic Iron Oxide Nanoparticles Conjugated with Folic Acid for Dual Target-Specific Drug Delivery and MRI in Cancer Theranostics. *Materials Science and Engineering: C* **2017**, *70*, 763–771. https://doi.org/10.1016/J.MSEC.2016.09.052.

(2) Bouché, M.; Hsu, J. C.; Dong, Y. C.; Kim, J.; Taing, K.; Cormode, D. P. Recent Advances in Molecular Imaging with Gold Nanoparticles. *Bioconjug Chem* **2019**, *31* (2), 303. https://doi.org/10.1021/ACS.BIOCONJCHEM.9B00669.

(3) Aghaei, M.; Nasimian, A.; Rahmati, M.; Kawalec, P.; Machaj, F.; Rosik, J.; Bhushan, B.; Bathaie, S. Z.; Azarpira, N.; Los, M. J.; Samali, A.; Perrin, D.; Gordon, J. W.; Ghavami, S. The Role of BiP and the IRE1α–XBP1 Axis in Rhabdomyosarcoma Pathology. *Cancers (Basel)* **2021**, *13* (19), 4927. https://doi.org/10.3390/CANCERS13194927/S1.

(4) Talarposhti, M. V.; Salehzadeh, A.; Jalali, A. Comparing the Toxicity Effects of Copper Oxide Nanoparticles Conjugated with Lapatinib on Breast (MDA-MB-231) and Lung (A549) Cancer Cell Lines. *Naunyn Schmiedebergs Arch Pharmacol* **2024**, *397* (9), 6855–6866. https://doi.org/10.1007/S00210-024-03071-1/METRICS.

(5) Raheem, M. A.; Rahim, M. A.; Gul, I.; Zhong, X.; Xiao, C.; Zhang, H.; Wei, J.; He, Q.; Hassan, M.; Zhang, C. Y.; Yu, D.; Pandey, V.; Du, K.; Wang, R.; Han, S.; Han, Y.; Qin, P. Advances in Nanoparticles-Based Approaches in Cancer Theranostics. *OpenNano* **2023**, *12*, 100152. https://doi.org/10.1016/J.ONANO.2023.100152.

(6) Deinavizadeh, M.; Kiasat, A. R.; Hooshmand, N.; Labouta, H. I.; Shafiei, M.; Sabaeian, M.; Mirzajani, R.; Zahraei, S. M.; Makvandi, P.; El-Sayed, M. A. Near-Infrared/PH Dual-Responsive Nanosponges Encapsulating Gold Nanorods for Synergistic Chemo-Phototherapy of Lung Cancer. *ACS Appl Nano Mater* **2023**, *6* (18), 16332–16342. https://doi.org/10.1021/ACSANM.3C02464/ASSET/IMAGES/LARGE/AN3C02464_0006.JPEG.

(7) Zhang, X.; Xu, B.; Ni, J.; Xiang, Y.; He, Z. Combined Chemo- and Photothermal Therapies of Non-Small Cell Lung Cancer Using Polydopamine/Au Hollow Nanospheres Loaded with Doxorubicin. *Int J Nanomedicine* **2024**, *19*, 9597–9612. https://doi.org/10.2147/IJN.S473137;WEBSITE:WEBSITE:TFOPB;PAGEGROUP:STRING:PUBLICATION.

(8) Daneshgar, H.; Bagherzadeh, M.; Sojdeh, S.; Safarkhani, M.; Edrisi, M.; Ojaghi, A.; Ahmadi, S.; Kiani, M.; Rabiee, N. Discovery of Valley-Hill Structures on the Surface of MOFs: Enhancing DOX Diffusion and Release through Nature-Made Channels. *Nano Materials Science* **2024**. https://doi.org/10.1016/J.NANOMS.2024.11.003.

(9) Ding, W.; Guo, L. Immobilized Transferrin Fe3O4@SiO2 Nanoparticle with High Doxorubicin Loading for Dual-Targeted Tumor Drug Delivery. *Int J Nanomedicine* **2013**, *8*, 4631. https://doi.org/10.2147/IJN.S51745.

(10) Ji, Z.; Yin, Z.; Jia, Z.; Wei, J. Carbon Nanodots Derived from Urea and Citric Acid in Living Cells: Cellular Uptake and Antioxidation Effect. *Langmuir* **2020**, *36* (29), 8632–8640. https://doi.org/10.1021/ACS.LANGMUIR.0C01598/SUPPL_FILE/LA0C01598_SI_001.PDF.
